# The degree of intratumor mutational heterogeneity varies by primary tumor sub-site

**DOI:** 10.18632/oncotarget.8448

**Published:** 2016-03-28

**Authors:** Levi G. Ledgerwood, Dhruv Kumar, Agda Karina Eterovic, Jo Wick, Ken Chen, Hao Zhao, Loubna Tazi, Pradip Manna, Spencer Kerley, Radhika Joshi, Lin Wang, Simion I. Chiosea, James David Garnett, Terance Ted Tsue, Jeremy Chien, Gordon B. Mills, Jennifer Rubin Grandis, Sufi Mary Thomas

**Affiliations:** ^1^ Department of Otolaryngology, University of Kansas Medical Center, and University of Kansas Cancer Center, Kansas City, MO, USA; ^2^ Department of Systems Biology and Bioinformatics, MD Anderson Cancer Center, Houston, TX, USA; ^3^ Department of Biostatistics, University of Kansas Medical Center, and University of Kansas Cancer Center, Kansas City, MO, USA; ^4^ Department of Computational Biology, MD Anderson Cancer Center, Houston, TX, USA; ^5^ Division of Biology, Kansas State University, Manhattan, KS, USA; ^6^ Physicians Reference Laboratory, Kansas City, MO, USA; ^7^ Department of Pathology, University of Pittsburgh and University of Pittsburgh Cancer Institute, Pittsburgh, PA, USA; ^8^ Department of Pathology, University of Kansas Medical Center, and University of Kansas Cancer Center, Kansas City, MO, USA; ^9^ Department of Cancer Biology, University of Kansas Medical Center, and University of Kansas Cancer Center, Kansas City, MO, USA; ^10^ Department of Otolaryngology, University of Pittsburgh and University of Pittsburgh Cancer Institute, Pittsburgh, PA, USA; ^11^ Department of Pharmacology and Chemical Biology, University of Pittsburgh and University of Pittsburgh Cancer Institute, Pittsburgh, PA, USA; ^12^ Department of Anatomy and Cell Biology, University of Kansas Medical Center, and University of Kansas Cancer Center, Kansas City, MO, USA

**Keywords:** HNSCC, HPV, intratumor heterogeneity, mutation, deep sequencing

## Abstract

In an era where mutational profiles inform treatment options, it is critical to know the extent to which tumor biopsies represent the molecular profile of the primary and metastatic tumor. Head and neck squamous cell carcinoma (HNSCC) arise primarily in the mucosal lining of oral cavity and oropharynx. Despite aggressive therapy the 5-year survival rate is at 50%. The primary objective of this study is to characterize the degree of intratumor mutational heterogeneity in HNSCC. We used multi-region sequencing of paired primary and metastatic tumor DNA of 24 spatially distinct samples from seven patients with HNSCC of larynx, floor of the mouth (FOM) or oral tongue. Full length, in-depth sequencing of 202 genes implicated in cancer was carried out. Larynx and FOM tumors had more than 69.2% unique SNVs between the paired primary and metastatic lesions. In contrast, the oral tongue HNSCC had only 33.3% unique SNVs across multiple sites. In addition, HNSCC of the oral tongue had fewer mutations than larynx and FOM tumors. These findings were validated on the Affymetrix whole genome 6.0 array platform and were consistent with data from The Cancer Genome Atlas (TCGA). This is the first report demonstrating differences in mutational heterogeneity varying by subsite in HNSCC. The heterogeneity within laryngeal tumor specimens may lead to an underestimation of the genetic abnormalities within tumors and may foster resistance to standard treatment protocols. These findings are relevant to investigators and clinicians developing personalized cancer treatments based on identification of specific mutations in tumor biopsies.

## INTRODUCTION

Head and neck squamous cell carcinoma (HNSCC) is the sixth most common cancer in the world, with nearly 600,000 new cases diagnosed annually [1]. Despite a vast array of research on HNSCC and development of new and less toxic treatment regimens, the survival rates of HNSCC have not dramatically changed over the last 50 years, with overall survival of approximately 50% [2]. This somewhat guarded outcome is, in part due to the development of chemotherapy and radiation resistance following an initial response, leading to locoregional and distant failures. A major factor contributing to this is the presence of intratumor genetic heterogeneity [3].

Great strides have been made in understanding the “mutational landscape” of head and neck squamous cell carcinoma (HNSCC) [4, 5]. With increasing incidence of HNSCC in younger patients, a recent report demonstrates that mutational frequencies in oral tongue tumors from young patients are similar to older patients [6]. The majority of HNSCC genomic studies have analyzed single samples from individual tumors, which does not allow for an understanding of intratumor heterogeneity. While the development of cancer is presumed to be due to clonal expansion of tumor cells [7], studies suggest that continued acquisition of mutations in the clones, leads to a heterogeneous population of cells within the tumor resulting in the development of selective resistances to therapies through Darwinian selection [8–11]. Understanding the genetic heterogeneity of tumors, as well as the evolution of further genetic alterations is of critical importance to the development of therapies aimed at eradicating all clones to successfully treat HNSCC. Two recent studies have also utilized next-generation sequencing technologies to demonstrate a high level of intratumor heterogeneity in HNSCC [3, 9]. Widespread intratumor heterogeneity was reported in specimens from different regions of the primary tumor and corresponding metastatic lymph nodes from a single patient with HNSCC [3]. Further, they reconstructed an evolution of these changes throughout the tumor clones evaluated, showing that the development of genetic alterations that were present in metastatic samples arose as late events. Mroz et. al., also demonstrated that a higher level of genetic heterogeneity portended a worse prognosis in HNSCC [9]. Overall, intratumor heterogeneity appears to be an important factor in both treatment and prognosis for HNSCC.

This concept of intratumor heterogeneity is also an important consideration as we move toward the era of personalized medicine [12]. Therapies that have been developed targeting specific antigens or markers on tumor cells rely on the ubiquity of expression of these markers on the tumor cell to have therapeutic value [13, 14]. Recently, the p.E322K mutation in *MAPK1* was reported to confer exquisite sensitivity to small molecule inhibitor erlotinib in a patient with tongue cancer [15]. The presence or absence of such markers is usually determined by single biopsies taken from tumors with the implicit assumption that expression of markers in the biopsy specimen is representative of the tumor as a whole. Recently, Gerlinger et al. demonstrated an intratuomor heterogeneity from single-biopsy sample of metastatic renal-cell carcinoma suggest that the distinct mutations in *mTOR, SETD2, PTEN*, and *KDM5C* genes can cause convergent phenotypic evolution of tumor [8]. However, it is a great challenge but understanding genomics landscape depicted from single tumor-biopsy samples may expose to the development of effective personalized-medicine and biomarker.

In this manuscript, we aimed to determine the degree of intratumor heterogeneity within both primary tumors and in metastatic lymph nodes among seven patients with oral tongue, FOM or laryngeal HPV negative HNSCC. We carried out deep sequencing of 202 genes implicated in cancer and validated the findings using the Affymetrix array platform and The Cancer Genome Atlas (TCGA) HNSCC dataset. Comparing oral tongue, FOM and laryngeal cancer specimens, we were able to show a greater level of intratumor genetic heterogeneity in the laryngeal tumors.

## RESULTS

### Patient characteristics

The patients ranged in age from 40 to 63 years, with a median of 57 years, five patients were male and two female (patient 4 and 7 with oral tongue and larynx tumors, respectively). All patients in the group were Caucasian (Supplemental Table 1). Six of seven patients had a history of smoking at the time of diagnosis, with only one patient having never smoked (patient 4 with a tongue tumor). Five of the seven patients had a history of alcohol consumption and all were still actively drinking at the time of diagnosis. Four of the seven patients (57.1%) had a primary tumor located in the oral tongue, one patient (14.3%) had an anterior floor-of-mouth primary tumor and two patients (28.6%) had supraglottic laryngeal primary squamous cell carcinoma. All patients were treatment naïve with the exception of patient 2. Patient 2 with a recurrent oral tongue tumor was previously treated with cisplatin and radiation therapy. Patient 7 had a new primary in the larynx. The first laryngeal SCC that occurred 10 y prior was an indolent T1N0 involving a different site of the larynx. The patient had no prior chemotherapy or radiation treatment. We tested the tumor samples for HPV positivity using immunohistochemistry, *in situ* hybridization and quantitative PCR. All samples were clinically negative for HPV infection. No evidence of HPV was identified with a sensitive real time qPCR assay capable of identifying the presence of 15 different serotypes of HPV (Supplemental Table 2).

### Degree of SNV heterogeneity varies by sub-site

**Figure 1:**
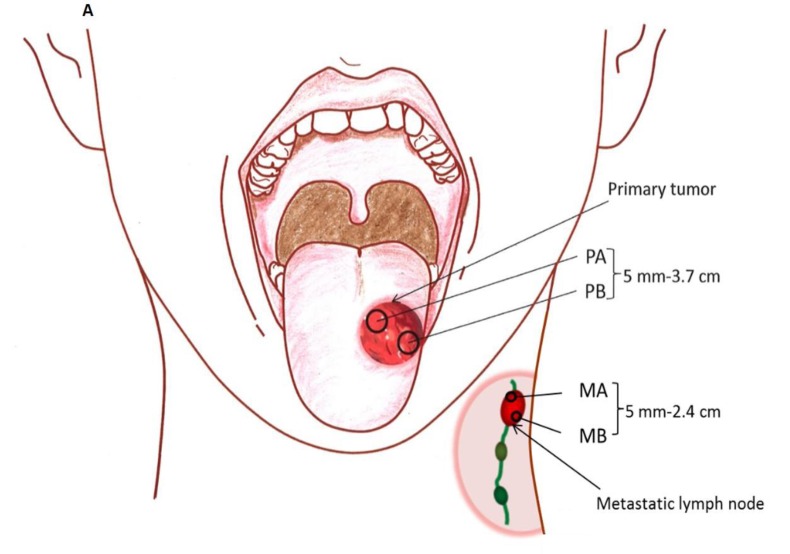

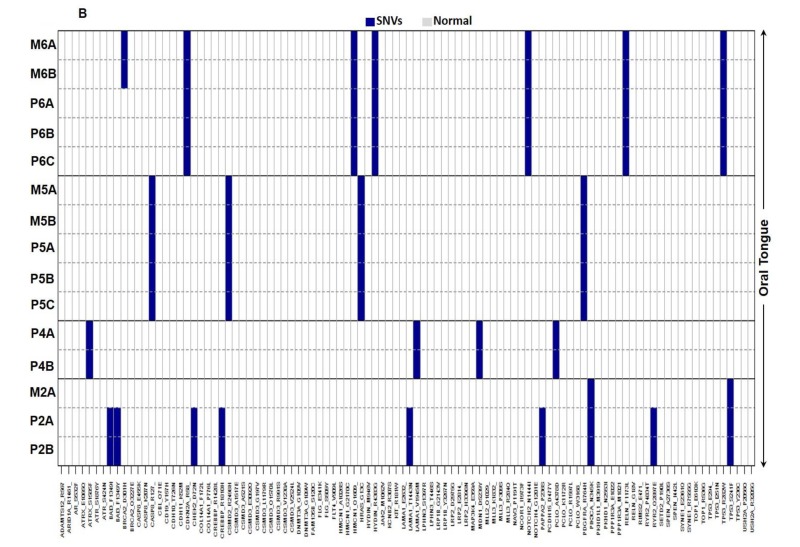

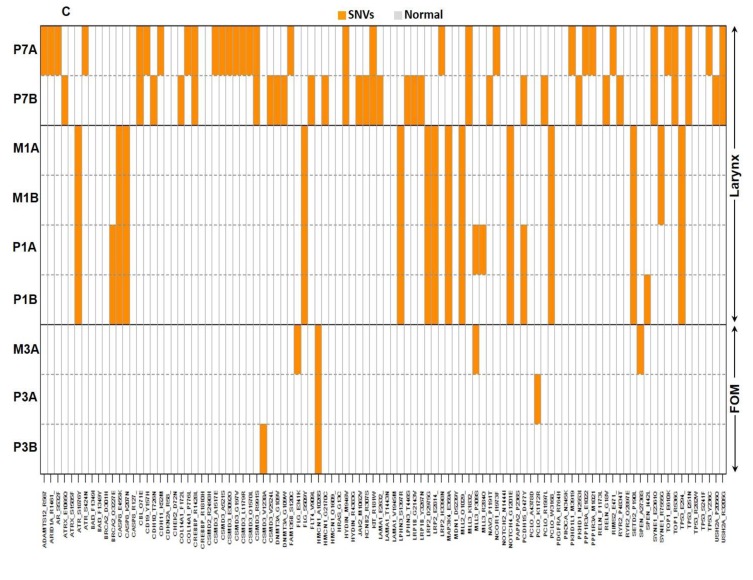

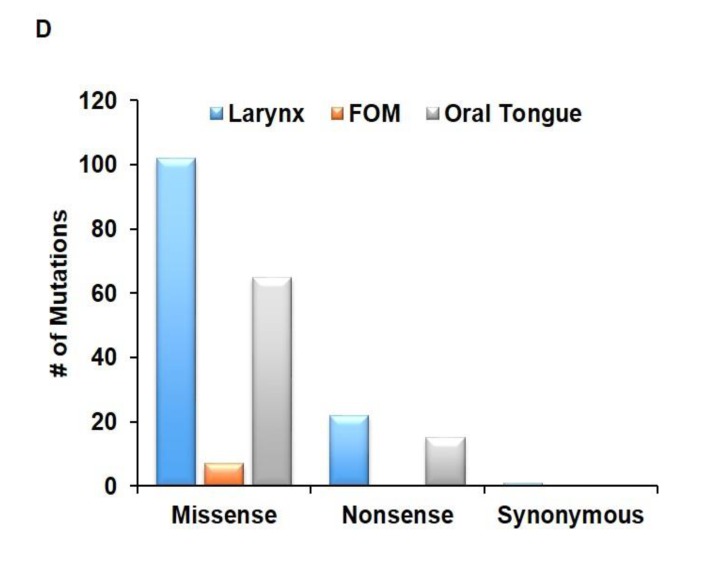
Degree of SNV heterogeneity varies based on sub-site **A.** Schematic depicting specimen acquisition from the primary (P) tumor or metastatic (M) lymph node. HNSCC specimens were taken from multiple locations separated by a distance of at least 5 mm at the primary tumor site and in most cases, paired metastatic lymph node. Figure demonstrating SNVs present (blue/orange) or absent (white) within primary (P) or metastatic (M) **B.** oral tongue HNSCC (patient 2, 4, 5 and 6), and **C.** laryngeal (patient 1 and 7) and FOM (patient 3) HNSCC. **D.** Functional consequences of SNVs present within tumor specimens are depicted in the bar graph. The majority of mutations from various sites were missense mutations.

Specimens were collected from 2-3 locations separated by at least 5 mm in the primary tumor (P) and in most cases, the matched metastatic lymph node (M) to evaluate the degree of intratumoral mutational heterogeneity (Figure 1A). Tumor content was assessed using H&E staining of specimens to confirm > 70% tumor by volume of specimen. Of 202 genes (*MTOR, NRAS, NOTCH2, FLG, IL6R, SPTA1, SPEN, DDR2, PAPPA2, HMCN1, USH2A, RYR2, ZNF238, ARID1A, CSMD2, MPL, JAK1, ERCC3, LRP1B, LRP2, ITGA4, CASP8, IDH1, PIKFYVE, IRS1, DNMT3A, ALK, SOS1, EML4, MSH2, MSH6, VHL, RAF1, FOXL2, ATR, MECOM, PIK3CA, ETV5, TGFBR2, MLH1, MYD88, CTNNB1, SETD2, PBRM1, BAP1, MITF, EPHA3, TET2, CRIPAK, FBXW7, FGFR3, WHSC1, PDGFRA, KIT, KDR, LPHN3, APC, CSF1R, PDGFRB, GABRA6, NPM1, FGFR4, NSD1, FLT4, CDH10, ADAMTS12, HEATR7B2, MAP3K1, PIK3R1, PRDM1, TNFAIP3, ESR1, SYNE1, MAP3K4, DDR1, NOTCH4, DAXX, PKHD1, BAI3, MDN1, RELN, PIK3CG, PPP1R3A, MET, SMO, BRAF, PRSS1, EZH2, MLL3, HDAC9, CARD11, IKZF1, EGFR, ELN, HGF, PCLO, CDK6, RIMS2, PKHD1L1, CSMD3, COL14A1, FAM135B, PTK2, CSMD1, FGFR1, KCNB2, RUNX1T1, PPP2R4, ABL1, TSC1, NOTCH1, CDKN2A, PAX5, JAK2, GNAQ, SYK, PTCH1, NFKB2, FGFR2, RET, PCDH15, GATA3, PTEN, CYP2C19, ATM, CBL, CHEK1, WT1, HRAS, MEN1, FAT3, PTPN11, HNF1A, KRAS, AKAP3, MLL2, ACVR1B, ERBB3, LRP1, CDK4, NAV3, ERCC5, FLT3, FLT1, BRCA2, RB1, TBC1D4, AKT1, SYNE2, TSHR, GABRB3, RAD51, MAP2K1, IDH2, IGF1R, ERCC4, TSC2, PALB2, CD19, CREBBP, CYLD, CDH11, CDH1, HYDIN, MAP2K4, NCOR1, NF1, HNF1B, ERBB2, TOP2A, BRCA1, SPOP, TP53, RNF213, AURKB, SMAD4, LAMA1, SMARCA4, STK11, NOTCH3, CPAMD8, JAK3, ZNF536, GNA11, CEBPA, TGFb1, PPP2R1A, ASXL1, TOP1, AURKA, GNAS, RUNX1, SMARCB1, CHEK2, NF2, EP300, KDM6A, ARAF, GATA1, FAM123B, AR and ATRX*) analyzed, 57 genes had a total of 102 somatic single nucleotide variations in at least one of the primary or metastatic sites across the seven patients (Supplemental Table 3). None of the SNVs were shared between patients. Of the SNVs detected, 71 were only seen in primary tumor specimens with 48 being present in only one sample from the primary tumor. Overall there was a much higher number of SNVs identified in laryngeal tumors (72 distinct SNVs) compared to oral tongue tumors (24 distinct SNVs).

When the degree of heterogeneity was evaluated based on sub-site, oral tongue tumors were found to have fewer mutations with only 33.3% of SNVs being distinct between specimens from the same patient compared to laryngeal tumors (Figure 1B). There was less heterogeneity between oral tongue tumors with 0/4, 0/4 and 1/7 unique SNVs in the 200 gene panel in patients 4, 5 and 6, respectively. Patient 2 with the previously treated recurrent oral tongue tumor demonstrated a total of 7/9 unique SNVs. Laryngeal and FOM tumors (Figure 1C) demonstrated higher level of intratumor heterogeneity compared to oral tongue tumors, showing 69.2% of SNVs which were not shared among all specimens from the same patient (χ^2^ (df=1) =5.02, exact p = 0.029).

The majority of SNVs conferred a missense mutation (82%) to the gene affected (Figure 1D). The number of missense mutations in laryngeal tumor specimens was not significantly different from oral tongue specimens (χ^2^ (df=1) = 0.7, exact p = 0.7). Patient 5 had somatic mutations in *HRAS* p.G13C, *CASP8* p.R127* and *PDGFRA* p.R764H at all sub-sites in primary and metastatic tumor samples again consistent with these being truncal events. The substitutions in *TP53* and *HRAS* have been previously reported in COSMIC consistent with these being driver mutations. The p.R127* substitution in CASP8 a protein critical in the initiation phase of apoptosis has not been reported; however, inactivating substitutions and frame-shift mutations in *CASP8* have been reported [16, 17]. Finally, p.R764H substitution in *PDGFRA* has not been previously reported; however, this substitution is within the kinase domain of PDGFRA (a.a. 593-948; PF07714).

**Figure 2:**
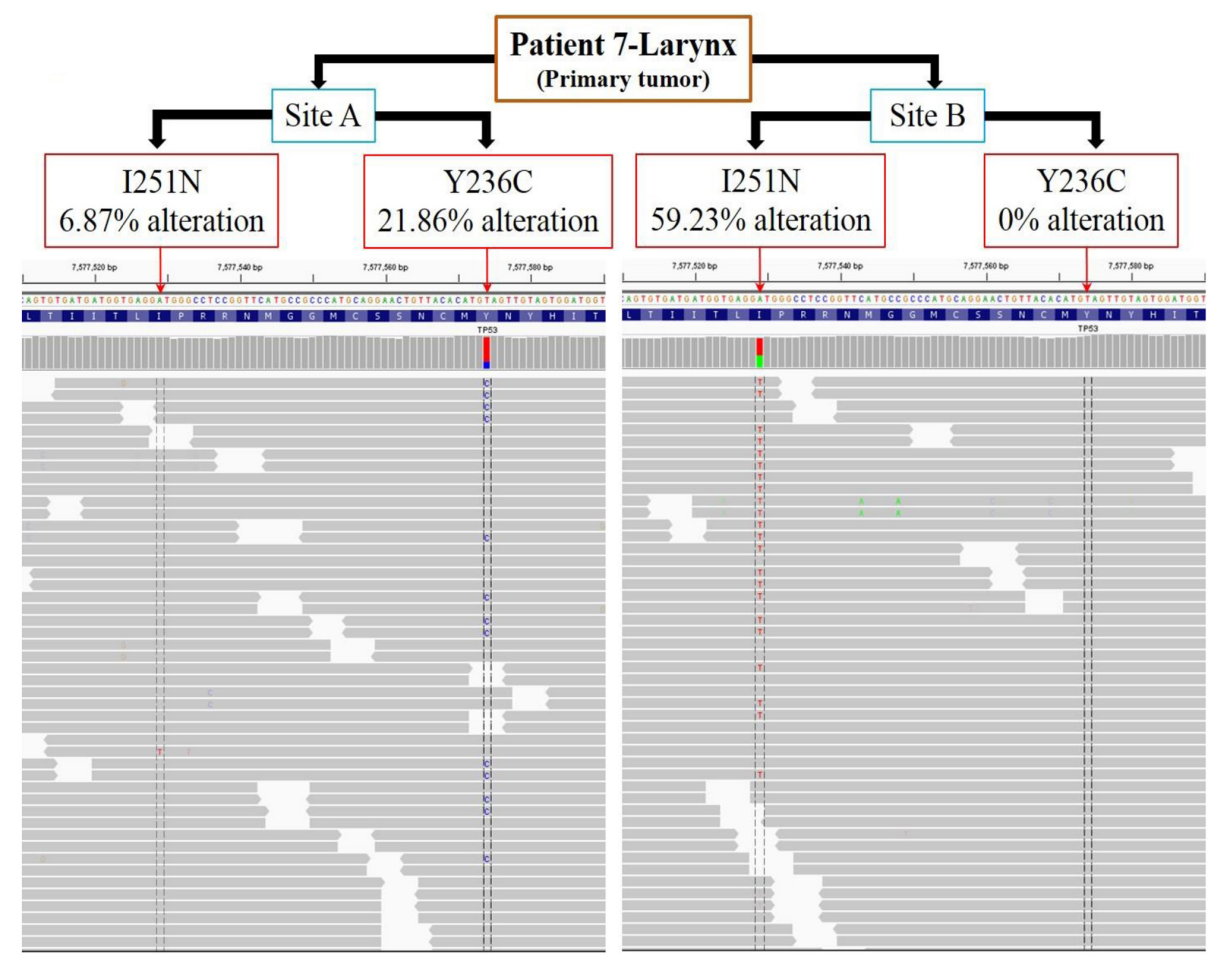
*TP53* mutations p.I251N and p.Y236C within the primary laryngeal tumor sub-sites developed independent of each other The primary tumor from patient 7 was assessed for mutations in *TP53* at 2 sites separated by a distance of 1 cm. Two specific mutations in *TP53* that were separated by 45 bases were analyzed in each sequencing read (gray bars). Across all sequencing reads, tumor A had 6.87% alterations in the amino acid residue p.I251N and 21.86% alterations in p.Y236C. Tumor B had 59.23% alterations in the p.I251N residue and no alteration in p.Y236C. Thus, mutations in p.I251N and p.Y236C residues emerged independently at different sites (A and B) within the primary tumor.

Two oral tongue tumors had somatic point mutations identified in *TP53* p.R282W in patient 6 and p.S241F in patient 2 in primary sub-sites and metastatic sub-sites consistent with this being a truncal event. In patient 7 (laryngeal tumor), a substitution at amino acid residue p.I251N in the *TP53* gene was observed in two sub-sites (A and B separated by a distance of 1 cm) of the primary tumor (Figure 2). Interestingly, another *TP53* mutation, p.Y236C separated by 45 bases from p.I251N, was unique to sub-site A. Further examination of each sequencing read from *TP53* fragments spanning the amino acid residues p.I251N and p.Y236C were carried out. We observed 21.86% alteration (T replaced by C) at amino acid residue p.Y236C and 6.87% alteration (A replaced by T) at p.I251N in the sub-site A. Further, at the tumor sub-site B, there was no alteration at p.Y236C and 59.23% alteration (A replaced by T) at p.I251N. Thus the p.I251N alteration at sub-site A developed independently of the p.Y236C alteration at tumor sub-site B. The number of nucleotide alterations in sub-site A and B laryngeal primary tumor from patient 7 were counted manually and the percentage calculated based on the Integrative Genomics Viewer (IGV) results.

### Copy number variations depend on the sub-site of origin

**Figure 3:**
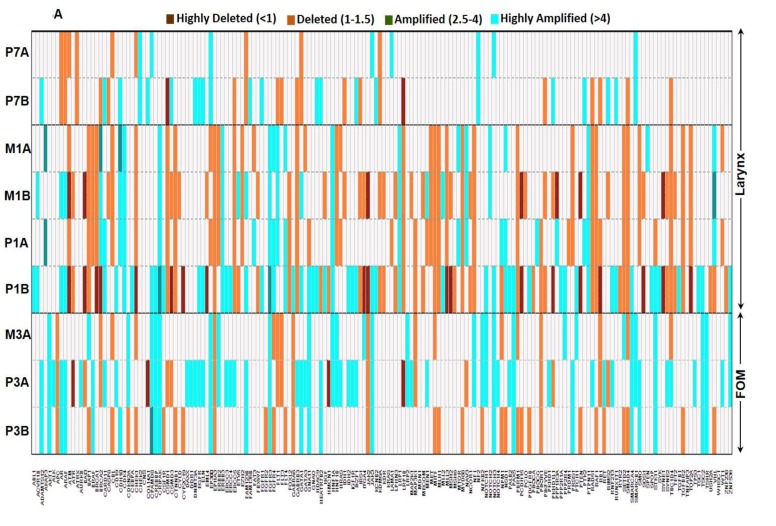

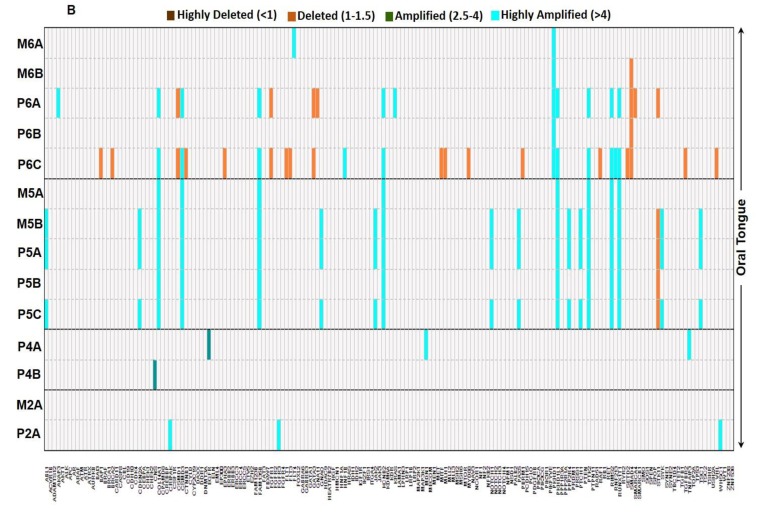
Heterogeneity in copy number varies based on the sub-site or origin The copy number variability identified within **A.** laryngeal (patient 1 and 7) and FOM (patient 3) tumors, and **B.** oral tongue tumors. The key specifies the color code to depict the status of CNVs in each gene. Larynx and FOM tumors demonstrated significantly higher number of CNVs compared to oral tongue tumors.

Frequently an amplification in the number of copies of a particular gene results in increased expression of the gene product contributing to the disease process. A total of 175 of the 202 genes (86.6%) demonstrated copy number variations (CNV) (Supplemental Table 4). Of the 175 genes with CNVs identified, 114 of these were shared between multiple patients. Of these CNVs (whether amplification, deletion, or both) 52 were shared between two patients, 50 were shared between three patients, nine shared between four patients, and three shared between five patients. There were 202 and 110 genes with CNVs in laryngeal and FOM tumors respectively, (Figure 3A). In contrast, oral tongue tumors demonstrated CNVs in a total of 62 genes (Figure 3B). A total of 22 genes demonstrated loss of heterozygosity (LOH). Larynx tumors had a higher number of genes with LOH (14 genes) compared to oral tongue tumors (8 genes).

### Phylogenetic analysis of mutations

**Figure 4:**
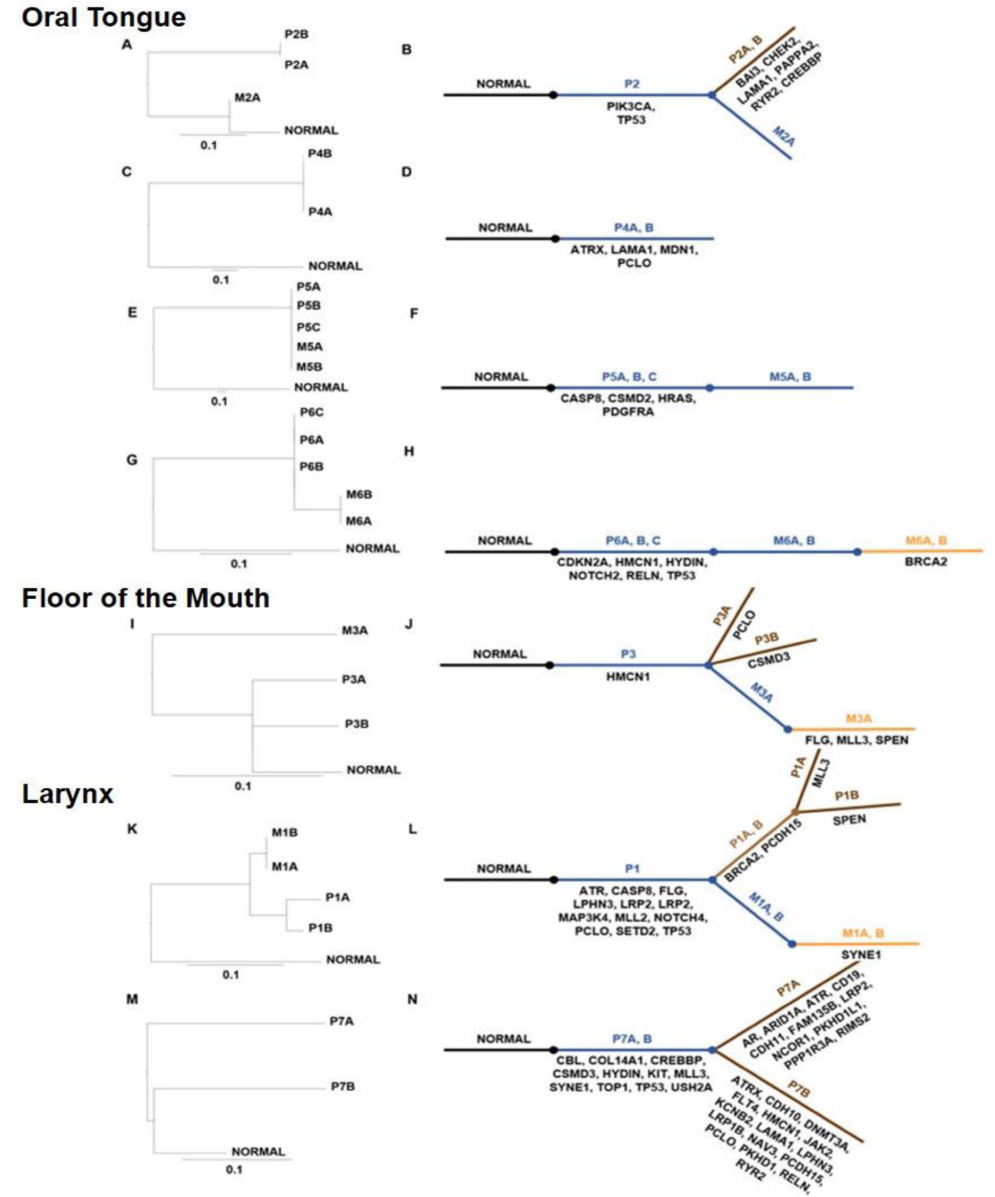
Evolutionary analyses of mutated codons in HNSCC from various sites Panels **A.**, **C.**, **E.**, **G.**, **I.**, **K.** and **M.** represent midpoint-rooted phylogenetic trees of the tumors using the maximum likelihood approach. The branch lengths are proportional to the number of nonsynonymous mutations between the branching points. The scale bar represents 0.1 substitutions per site. Panels **B.**, **D.**, **F.**, **H.**, **J.**, **L.** and **N.** represent the evolutionary models of tumors in each patient and list the mutated genes in each cluster. The commonly mutated genes are in dark blue and genes with unique mutations are listed below the brown and orange lines for the primary and metastatic tumor, respectively.

Phylogenetic trees were generated on clusters of mutations (e.g. Missense, Nonsense and Synonymous) to determine the clonal evolution of tumors relative to the germline non-mutated sequence (Figure 4A–4N). In addition, we listed the unique and commonly mutated gene names in an evolutionary model of these tumors. The length of the trunk and branches on phylogenic trees denote the number of mutations in that lineage. Mutated genes that are common between the primary and metastatic tumors are listed in the trunk below (blue). Unique mutations that evolve in the primary or metastatic tumor are listed in the branches colored brown and orange, respectively. For instance, in patient 3, *HMCN1* is commonly mutated in all specimens and hence listed next to the trunk. Unique mutations in *PCLO* and *CSMD3* in P3A and P3B, respectively and *FLG*, *MLL3* and *SPEN* in metastatic tumor M3A are listed in the branches. The metastatic tongue tumor from patient 2 was phylogenetically closer to the normal than the primary tumor. This indicated that P2A, P2B and M2A evolved from a precursor primary tumor with mutations in *TP53* and *PIK3CA* genes. Similarly, metastatic tumors in patient 1 were phylogenetically closer to the normal compared to the primary laryngeal tumor indicating that all these tumors originated from an unsampled tumor that subsequently acquired additional mutations detected in P1A and P1B. The laryngeal tumor from patient 7 demonstrates a high degree of mutations in P7A and P7B specimens indicating a high level of intratumoral heterogeneity.

### Affymetrix and TCGA analysis validates deep sequencing data of HNSCC

**Figure 5:**
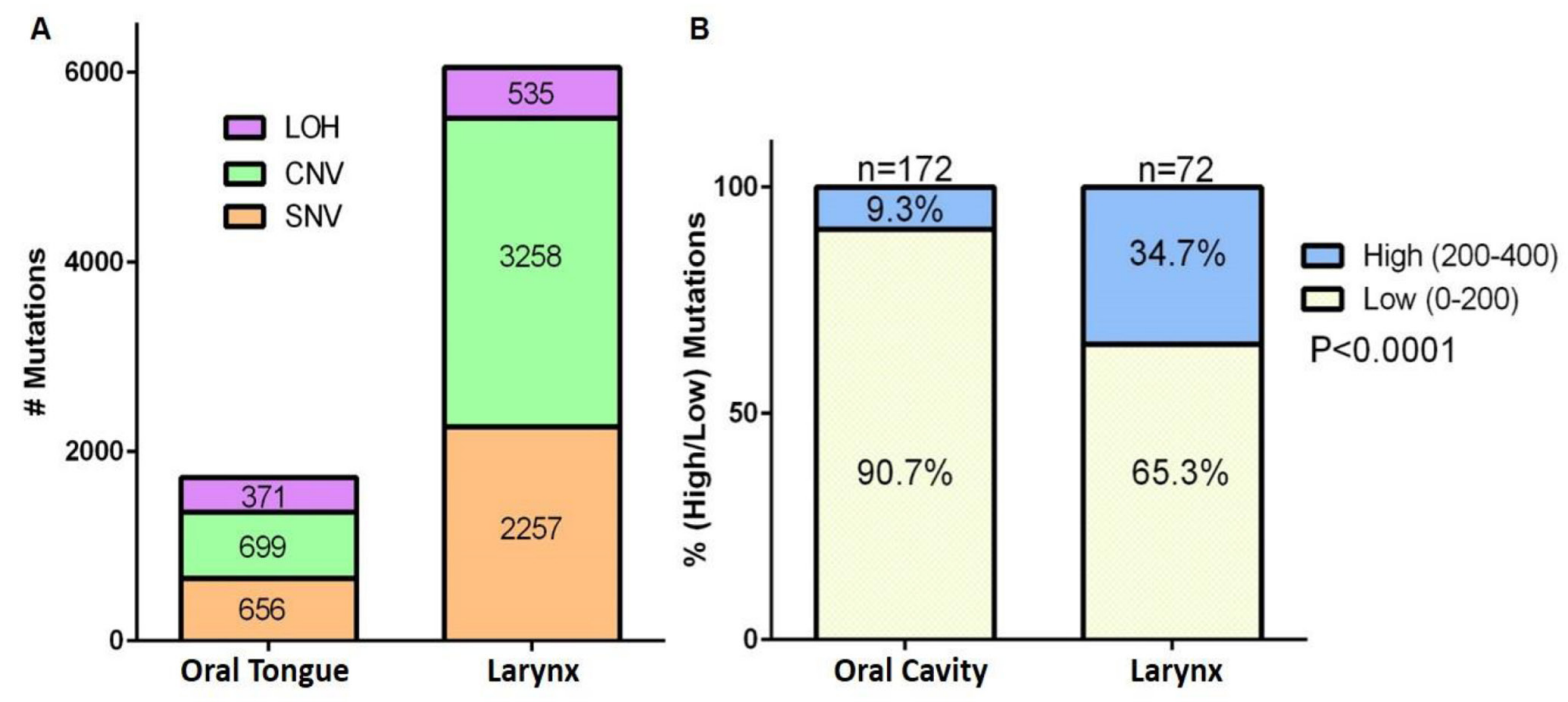
Affymetrix SNP 6.0 array and TCGA data analysis demonstrate that oral cavity tumors have fewer mutations than larynx tumors **A.** Number of single nucleotide variations (SNV), copy number variations (CNV) and loss of heterozygosity (LOH) in all samples from patient 1 (larynx tumor) and patient 5 (oral tongue tumor) are graphed. Numbers in the stacked bars indicate the number of mutations. **B.** TCGA analyses of percentage of genes with low (0-200) or high (200-400) number of mutations in tumor from oral cavity or larynx.

Affymetrix whole genome SNP array data obtained from oral tongue (patient 5) and larynx (patient 1) tumors were analyzed using the Genotyping Console Software 4.2. Analysis of the SNV, CNV, and LOH confirmed a significantly higher rate of alterations in the laryngeal tumors as compared to oral tongue tumors (χ^2^ (df=2) = 231.7, exact p < 0.001) (Figure 5A). Further, analyses of The Cancer Genome Atlas (TCGA) data revealed that oral cavity tumors have significantly fewer mutations than laryngeal tumors (χ^2^ (df=1) = 23.45, exact p < 0.0001) (Figure 5B).

## DISCUSSION

While the development of cancer is presumed to be due to clonal expansion of tumor cells [7], studies suggest that during this expansion there is continued acquisition of mutations in the clones, leading to a heterogeneous population of cells within the tumor [8–11]. Recent work on renal cell carcinoma and pancreatic carcinoma have demonstrated significant intratumor heterogeneity, and suggest that metastatic potential of these tumors may develop late in their evolution and from only a small subset of the tumor cells [8, 18]. The maintenance of these heterogeneous populations within the tumor can lead to the development of selective resistance to therapies through Darwinian selection [8, 10]. Our evaluation of HNSCC specimens from seven patients shows that intratumor heterogeneity in HNSCC varies based on the sub-site in which the tumor arises. All tumors evaluated had varying degrees of mutational heterogeneity when evaluating both SNVs and CNVs between tumor specimens. This is the first report to demonstrate that intratumor heterogeneity in HNSCC varies by primary site of the tumor in question.

Stransky et al. demonstrated in a well-designed study of 92 patients that a large number of genes are altered in HNSCC, with a large portion of these genes involved in squamous differentiation [4]. Many mutations in a wide array of genes related to epithelial differentiation were found, however they also noted that there seemed to be unifying features of HNSCC despite the sub-site of origin in the tumors they evaluated. As an example, *TP53* in their study was found to be inactivated in nearly all patients evaluated. This study was not designed to evaluate intratumor heterogeneity, as samples used were from single biopsy specimens from each patient. *TP53* mutations were similarly identified in the current study; however, these mutations were heterogeneously present within the specimens evaluated. While studies demonstrating the various mutations present throughout the genome of HNSCC cases are of importance, it remains crucial to take into account the presence of intratumor heterogeneity in treatment planning.

Mroz et al. utilized a process they developed deemed mutant-allele tumor heterogeneity (MATH) to evaluate heterogeneity in tumor specimens [9]. Through this technique, they determined that increased levels of heterogeneity within the tumors depending on HPV status conferred a poorer prognosis for HNSCC. Further, the authors demonstrated that the degree of heterogeneity was more strongly associated with poor prognosis than either HPV status of the tumor or the presence of a deleterious mutation in the *TP53* gene. In a more recent application of MATH analyses to the TCGA cohort, Mroz et al reported that high MATH values indicative of increased heterogeneity correlated with reduced overall survival [19]. It would be interesting to correlate patient survival to the extent of mutations in the tumor. Our data demonstrate that direct evaluation of multiple specimens from HNSCC tumors reveals an underappreciated difference in the number of mutations based on site.

Various differences between the larynx, FOM and the oral tongue may account for the disparity of incidence in mutations at these sites. The anterior two-thirds [20] of the tongue and the floor-of-mouth arise from embryological arches 1 and 2, while the larynx develops from arches 3 and 4. The oral cavity is made up of pseudostratified epithelium to be more resilient to trauma during mastication, while the larynx has respiratory epithelium and is primarily exposed to air. The FOM is a horseshoe-shaped region beneath the tongue. HNSCC in the FOM can demonstrate rapid progression that is difficult to control locally and regionally. The most likely cause for the differences in mutation rates between various sites could be the extent of carcinogen exposure at these sites. The oral tongue has a larger surface area compared to the larynx. Further, the larynx is exposed to carcinogens during both inhalation and exhalation. As air flows over the larynx, eddy currents created by the false vocal cords increases exposure to the region. The increased exposure to carcinogen could account for an increase in mutation rates in tumors of the larynx compared to the oral tongue.

This study provides evidence that the degree of intratumor heterogeneity in squamous cell carcinoma varies by location of the primary tumor. These data demonstrate that, although multi region sampling is needed to fully assess intratumor heterogeneity, single-biopsy may adequately represent mutations in oral cavity tumors. Studies in much larger cohorts with comprehensive clinical annotation and repeat biopsy at relapse are needed to fully understand the clinical relevance of intratumor heterogeneity in head and neck cancer. Moreover, detail study of epigenetic and phenotypic evaluation through DNA methylation, chromatin remodeling, RNA and protein expression studies are needed to fully understand the impact of intratumor heterogeneity on tumor biology and response to therapy.

## MATERIALS AND METHODS

### Sample collection and genomic DNA preparation

Research involving collection of tissues and clinical information from human subjects was approved by the Institutional Review Board of the University of Pittsburgh, and informed consent was obtained from each patient prior to tissue collection. Patients undergoing resection of HNSCC were considered for inclusion in this study. Seven patients with HNSCC were included in the study. In each case, specimens were collected from at least two separate locations of the primary tumor and in most cases from two separate locations of a metastatic lymph node. These samples were separated by at least 5 mm, with a range of 5-37 mm between samples (Supplemental Table 5). Details of patient's tumor tissues information are summarized in Supplemental Table 5. Blood samples were also collected from patients to evaluate the genomic DNA from lymphocytes as controls for germ line genetic alterations in each patient. Blood samples were centrifuged and the “buffy coat” was harvested for collection of lymphocytes. Tissue specimens were embedded in Tissue-Tek OCT compound (Sakura Finetek, Torrance, CA), sectioned at 20 μm thickness (12 sections per sample) and collected in tubes for DNA extraction. One 8 μm section was collected before and after the 20 μm sections for histological analyses of hematoxyllin and eosin stained sections to ensure presence of > 70% tumor cells. DNA was extracted using the Qiagen DNA mini kit (Valencia, CA), following manufacturer's instructions. DNA was quantified using PicoGreen dsDNA Quantitation Reagent (Invitrogen, Carlsbad, CA).

### HPV testing

HPV infection status in tissues was determined as previously described using FISH and multi-plex PCR [21, 22]. For quantitative PCR analyses briefly, HPV testing was performed on OCT embedded tissue samples using the COMPLeTe Care HPV test (Physicians Reference Laboratory, LLC, Overland Park, KS), a multiplex real-time PCR test that simultaneously detects, types, and quantifies all 15 high-risk HPV types known to cause anogenital cancer. Five to 10 tissue sections (5 μm each) were used to extract DNA using a QIAmp tissue kit (Qiagen, Valencia, CA). Four multiplex reactions, each targeting four high-risk HPV types, were performed in a LightCycler 480 instrument using 8 μL of extracted DNA per multiplex reaction to detect all 15 high-risk HPV types and an internal control in which HPV 16 was detected in two multiplex reactions. Quantitative standards and controls for each of the high-risk types and internal control β-globin were included in each run. The high-risk HPV subtypes tested were 16, 18, 31, 33, 35, 39, 45, 51, 52, 56, 58, 59, 68, 73, and 82, chosen according to a consensus from several epidemiologic studies. The COMPLeTe Care HPV test targets E7 of the HPV genome, an oncogene, the presence of which is required for oncogenesis. Unlike capsid gene L1, which is reported to be lost on integration of the HPV genome into the host chromosome, the oncogenes E6 and E7 are stable. The test also detects and quantifies the β-globin (HBB) gene as an internal control.

### Genomic DNA library preparation

Genomic DNA was quantified by Qubit (Invitrogen, Carlsbad, CA) and quality was accessed using Genomic DNA Tape for the 2200 Tapestation (Agilent Technologies, Santa Clara, CA). DNA from each sample (200-500 ng of genomic DNA) was sheared by sonication with the following conditions: Peak Incident Power 175, Duty Cycle 20%, Intensity 5, Cycles per Burst 200, and 120 seconds using Covaris E220 instrument (Covaris Inc., Woburn, MA). To ensure the proper fragment size, sonicated DNA samples were checked on TapeStation using the DNA High Sensitivity kit (Agilent Technologies, Santa Clara, CA). The sheared DNA proceeded to library prep using KAPA library prep kit (KAPA) following the “with beads” manufacturer protocol. Briefly, this protocol consists of 3 enzymatic reactions for end repair, A-tailing and BioO adaptor ligation, followed by barcode (BioO Scientific, Austin, Texas) insertion by PCR using KAPA HiFi polymerase (6 cycles). PCR primers were removed by using 1.8x volume of Agencourt AMPure PCR Purification kit (Beckman Coulter, Brea, CA). At the end of the library prep, samples were analyzed on TapeStation to verify correct fragment size and to ensure the absence of extra bands. Samples were quantified using KAPA qPCR quantification kit. Equimolar amounts of DNA were pooled for capture (8-12 samples per pool).

### Targeted capture and deep sequencing

A total of 202 genes that are clinically relevant in cancer were selected for capture (Supplemental Table 6). The selection was based on mutational data in the Catalogue of Somatic Mutations in Cancer (COSMIC) and The Cancer Genome Atlas (TCGA) with a minimum of 3% frequency across disease sites or 5% disease specific frequency. We designed biotin labeled probes with Roche Nimblegen (Roche NimbleGen, Madison, WI) for capturing target regions (all exons in those 202 genes) and followed manufacture's protocol for the capture step. Briefly, DNA was pooled (8-16 samples), dried out and after addition of the capture reagents and probes, samples were incubated at 47°C on thermocycler with heated lid (57°C) for 64-74 hours. The targeted regions were recovered using streptavidin beads and the streptavidin-biotin-probe-target complex was washed and another round of PCR amplification was performed according to manufacturer's protocol. The quality of each captured sample was analyzed on TapeStation using the DNA High Sensitivity kit and the enrichment was accessed by qPCR using specific primers designed by Roche Nimblegen. The cutoff for the enrichment was 50 fold minimum.

The captured libraries were sequenced on a HiSeq 2000 (Illumina Inc., San Diego, CA) on a version 3 TruSeq paired end flowcell according to manufacturer's instructions at a cluster density between 700 - 1000 K clusters/mm^2^. Sequencing was performed on a HiSeq 2000 for 2 × 100 paired end reads with a 7 nucleotide read for indexes using Cycle Sequencing v3 reagents (Illumina Inc., San Diego, CA). The resulting BCL files containing the sequence data were converted into “.fastq.gz” files and individual libraries within the samples were demultiplexed using CASAVA 1.8.2 with no mismatches. All regions were covered by >20 reads. Average depth of sequencing was 1135x across the T200 exons. Most of mutations (202/212) was supported by at least 500 reads, 10 was supported by at least 98 reads. Five of the ten mutations that did not reach 500x were from CDKN2A, due to a very high GC content at this site. Having a minimal 500x coverage ensures confident (<2% FDR) sensitive (>95% chance) detection of low frequency (1%) mutations.

### SNV, CNV and LOH analysis

We aligned the T200 target-capture deep-sequencing data to human reference assembly hg19 using the Burrows-Wheeler Alignment (BWA) tool [23] and removed duplicated reads using samtools [24]. We called single nucleotide variants (SNVs) and small indels using VarScan2 [25], which classified variants into 3 categories: somatic, germline, and loss of heterozygosity based on the difference of allele frequencies between the tumor and the matched normal tissues. To ensure specificity, variants with an allele frequency less than 5% were not reported. We called copy number alterations using a previously published algorithm [26], which reports gain or loss status of each exon. To understand the potential functional consequence of detected variants, we compared them with dbSNP, COSMIC [27], and TCGA databases, and annotated them using VEP [28], Annovar [29], SIFT [30], Polyphen [31], Condel [32], Mutation Assessor [11] and CanDrA (Mao et al, unpublished).

### Microarray analysis

Processing of the Genome-Wide Human SNP 6.0 arrays was performed using the Affymetrix GeneChip (Affymetrix p/n 901182) at the KUMC Microarray Facility. The labeled target was prepared using the SNP 6.0 Core Reagent Kit (Affymetrix p/n 901706) according to the Affymetrix Genome-Wide Human SNP Nsp/Sty 6.0 User Guide rev.7 (Affymetrix p/n 702504). The target preparation was initiated with the restriction digestion of separate 250 ng aliquots of each genomic DNA using Nsp I and Sty I restriction endonuclease. The individual restricted fragments underwent ligation with the appropriate Nsp or Sty adaptor containing a PCR Primer 002 priming site. The ligated samples are diluted and PCR amplified using the PCR Primer 002 and Titanium DNA Amplification Kit (Clontech p/n 639240/639243). The Nsp amplification was performed in quadruplicate and the Sty amplification was performed in triplicate. The Nsp and Sty amplified fragments for each sample were pooled and purified using the Isopropanol Precipitation Method. Following quantification, the combined Nsp and Sty purified target samples were fragmented with DNase I and end labeled with biotin using Terminal Deoxynucleotidyl Transferase (TdT) and DNA labeling reagent. Biotin labeled-fragmented single stranded cDNA was hybridized to the Genome-Wide Human SNP 6.0 arrays according to manufacturer's instructions. Hybridized, washed and R-phycoerythrin-streptavidin stained arrays were scanned using the GeneChip Scanner 3000 7G with autoloader. Data collection was performed using the Command Console software (Affymetrix, Santa Clara, CA). Genotyping Console Software 4.2 (GTC) was used to analyze the copy number variation (CNV) and loss of heterozygosity (LOH) in five samples obtained from the patient with the larynx tumor and six samples obtained from the patient with an oral tongue tumor with regional GC correction.

### Phylogenetic tree construction and tumor evolution

Phylogenetic trees were generated to represent the intratumor heterogeneity and mutation evolutionary patterns and were estimated using the maximum likelihood approach [33], as implemented in PhyML [34]. Tumor samples were segregated by clusters of common and unique mutations in primary and metastatic sites. Synonymous and non-coding mutations were included in the analyses. An evolutionary model for each patient lists the genes with common and unique mutations in primary and metastatic tumors. Genes that acquired a common mutation during evolution in both primary and metastatic tumors are indicated next to normal (dark blue), and branches indicate unique mutations present in primary and metastatic sites (brown and orange, respectively).

### TCGA analysis

Somatic mutation data were downloaded from the public TCGA data repository website, cBioportal.org, from the Memorial Sloan Kettering Cancer Center. Data from 172 oral cavity and 72 larynx SCC patients were included in the analysis. The number of patients with low (</=200) and high (>200) mutation counts were determined.

### Statistical analysis

Due to the small sample size, medians and ranges are reported for continuous patient and tumor characteristics. Frequencies and percentages are reported for categorical characteristics. Chi-square tests were used to investigate associations between mutation rates and primary tumor location (oral cavity versus larynx). Exact p-values are reported. The Frequency Procedure in SAS version 9.4 was used. For TCGA datasets, the chi-square analysis was performed to determine if dichotomous mutation levels are associated with the site of tumor.

## SUPPLEMENTARY MATERIALS TABLES



## Abbreviations

HNSCC: head and neck squamous cell carcinoma
CNV: copy number variation
SNV: single nucleotide variant
SNP: single nucleotide polymorphism
LOH: loss of heterozygosity
HPV: human papilloma virus
TCGA: the cancer genome atlas.
