# Supplementary material for: The degree of intratumor mutational heterogeneity varies by primary tumor sub-site
Source: Oncotarget. 2016 Mar 28;7(19):27185–98. doi: 10.18632/oncotarget.8448 (PMC5053641; doi:10.18632/oncotarget.8448)
Supplement: Supplementary file 1 [file oncotarget-07-27185-s001.pdf]

The degree of intratumor mutational heterogeneity varies by primary tumor sub-site

Supplementary Material

Supplementary Table 1: Summary of HNSCC patient characteristics.

Characteristic	HNSCC Cases (N=7)
Age, years	
Median (range)	57 (40-63)
Sex, N (%)	
Men	5 (71.4)
Women	2 (28.6)
Smoking, N (%)	
Never	1 (14.3)
Former Smoker	5 (71.4)
Active Smoker	1 (14.3)
Alcohol , N (%)	
Never	2 (28.6)
Ever	5 (71.4)
Cancer Site, N (%)	
Oral cavity	5 (71.4)
Larynx (Supraglottic)	2 (28.6)
Cancer Type, N (%)	
Primary	6 (85.7)
Recurrence	1 (14.3)
Tumor Path Stage, N (%)	
T1-T2	1 (14.3)
T3-T4	5 (71.4)
Unstaged recurrent tumor	1 (14.3)
Nodal Path Stage, N (%)	
N0-N1	2 (28.6)
N2-N4	5 (71.4)
Perineural Invasion, N (%)	
Yes	4 (57.1)
No	3 (42.9)
HPV, N (%)	
Positive	0 (0.0)
Negative	7 (100)
P16, N (%)	
Positive	0 (0.0)
Negative	7 (100)

Supplementary Table 2: Tumors examined were HPV negative. HPV subtypes were assessed using specific primer with qRT-PCR. Positive controls for each subtype were included in the assay. Ct values for the positive control and β-globin are included.

Sample name	Site	HPV 16	HPV 18	HPV 31	HPV 33	HPV 39	HPV 45	HPV 51	HPV 52	HPV 56	HPV 58	HPV 59	HPV 68	HPV 73	HPV 82	β-Globin (Ct)
P1	Larynx	-	-	-	-	-	-	-	-	-	-	-	-	-	-	27.88
P2	Tongue	-	-	-	-	-	-	-	-	-	-	-	-	-	-	27.57
P3	Floor-of-mouth	-	-	-	-	-	-	-	-	-	-	-	-	-	-	27.24
P4	Tongue	-	-	-	-	-	-	-	-	-	-	-	-	-	-	27.19
P5	Tongue	-	-	-	-	-	-	-	-	-	-	-	-	-	-	27.44
P6	Tongue	-	-	-	-	-	-	-	-	-	-	-	-	-	-	27.19
P7	Larynx	-	-	-	-	-	-	-	-	-	-	-	-	-	-	27.38
Positive Control		29.6	28.7	28.2	27.6	29.9	28.7	28.9	27.6	27.4	27.1	28.1	28	30.2	29.3	26.47

Supplementary Table 3: SNVs in oral tongue, FOM and laryngeal tumors.

**Oral Tongue**					
**Gene Name**	**Sample**	**Variation**	**Protein position**	**Amino acids**	**Consequence**
ATRX	P4A	X_76890140_C/T	1585	S/F	non_synonymous_codon
ATRX	P4B	X_76890140_C/T	1585	S/F	non_synonymous_codon
BAI3	P2A	6_70071210_T/A	1349	F/I	non_synonymous_codon
BAI3	P2A	6_70071211_T/A	1349	F/Y	non_synonymous_codon
BAI3	P2B	6_70071210_T/A	1349	F/I	non_synonymous_codon
BAI3	P2B	6_70071211_T/A	1349	F/Y	non_synonymous_codon
BRCA2	M6A	13_32906516_G/C	301	D/H	non_synonymous_codon
BRCA2	M6B	13_32906516_G/C	301	D/H	non_synonymous_codon
CASP8	M5A	2_202131411_C/T	127	R/*	stop_gained
CASP8	M5B	2_202131411_C/T	127	R/*	stop_gained
CASP8	P5A	2_202131411_C/T	127	R/*	stop_gained
CASP8	P5B	2_202131411_C/T	127	R/*	stop_gained
CASP8	P5C	2_202131411_C/T	127	R/*	stop_gained
CDKN2A	M6A	9_21971186_C/T	58	R/*	stop_gained
CDKN2A	M6B	9_21971186_C/T	58	R/*	stop_gained
CDKN2A	P6A	9_21971186_C/T	58	R/*	stop_gained
CDKN2A	P6B	9_21971186_C/T	58	R/*	stop_gained
CDKN2A	P6C	9_21971186_C/T	58	R/*	stop_gained
CHEK2	P2A	22_29099524_G/A	72	D/N	non_synonymous_codon
CHEK2	P2B	22_29099524_G/A	72	D/N	non_synonymous_codon
CREBBP	P2A	16_3779619_G/A	1810	R/H	non_synonymous_codon
CREBBP	P2B	16_3779619_G/A	1810	R/H	non_synonymous_codon
CSMD2	M5A	1_34042979_G/A	2498	R/H	non_synonymous_codon
CSMD2	M5B	1_34042979_G/A	2498	R/H	non_synonymous_codon
CSMD2	P5A	1_34042979_G/A	2498	R/H	non_synonymous_codon
CSMD2	P5B	1_34042979_G/A	2498	R/H	non_synonymous_codon
CSMD2	P5C	1_34042979_G/A	2498	R/H	non_synonymous_codon
HMCN1	M6A	1_185984485_C/T	1609	Q/*	stop_gained
HMCN1	M6B	1_185984485_C/T	1609	Q/*	stop_gained
HMCN1	P6A	1_185984485_C/T	1609	Q/*	stop_gained
HMCN1	P6B	1_185984485_C/T	1609	Q/*	stop_gained
HMCN1	P6C	1_185984485_C/T	1609	Q/*	stop_gained
HRAS	M5A	11_534286_G/T	13	G/C	non_synonymous_codon
HRAS	M5B	11_534286_G/T	13	G/C	non_synonymous_codon
HRAS	P5A	11_534286_G/T	13	G/C	non_synonymous_codon
HRAS	P5B	11_534286_G/T	13	G/C	non_synonymous_codon
HRAS	P5C	11_534286_G/T	13	G/C	non_synonymous_codon

Gene Name	Sample	Variation	Protein position	Amino acids	Consequence
HYDIN	M6A	16_70871688_C/G	4383	R/G	non_synonymous_codon
HYDIN	M6B	16_70871688_C/G	4383	R/G	non_synonymous_codon
HYDIN	P6A	16_70871688_C/G	4383	R/G	non_synonymous_codon
HYDIN	P6B	16_70871688_C/G	4383	R/G	non_synonymous_codon
HYDIN	P6C	16_70871688_C/G	4383	R/G	non_synonymous_codon
LAMA1	P2A	18_7002317_C/A	1443	T/N	non_synonymous_codon
LAMA1	P2B	18_7002317_C/A	1443	T/N	non_synonymous_codon
LAMA1	P4A	18_6982553_G/A	1945	V/M	non_synonymous_codon
LAMA1	P4B	18_6982553_G/A	1945	V/M	non_synonymous_codon
MDN1	P4A	6_90362821_G/T	5239	D/Y	non_synonymous_codon
MDN1	P4B	6_90362821_G/T	5239	D/Y	non_synonymous_codon
NOTCH2	M6A	1_120468108_A/T	1444	N/I	non_synonymous_codon
NOTCH2	M6B	1_120468108_A/T	1444	N/I	non_synonymous_codon
NOTCH2	P6A	1_120468108_A/T	1444	N/I	non_synonymous_codon
NOTCH2	P6B	1_120468108_A/T	1444	N/I	non_synonymous_codon
NOTCH2	P6C	1_120468108_A/T	1444	N/I	non_synonymous_codon
PAPPA2	P2A	1_176526164_C/T	236	P/S	non_synonymous_codon
PAPPA2	P2B	1_176526164_C/T	236	P/S	non_synonymous_codon
PCLO	P4A	7_82544169_C/A	4378	A/D	non_synonymous_codon
PCLO	P4B	7_82544169_C/A	4378	A/D	non_synonymous_codon
PDGFRA	M5A	4_55146617_G/A	764	R/H	non_synonymous_codon
PDGFRA	M5B	4_55146617_G/A	764	R/H	non_synonymous_codon
PDGFRA	P5A	4_55146617_G/A	764	R/H	non_synonymous_codon
PDGFRA	P5B	4_55146617_G/A	764	R/H	non_synonymous_codon
PDGFRA	P5C	4_55146617_G/A	764	R/H	non_synonymous_codon
PIK3CA	M2A	3_178921553_T/A	345	N/K	non_synonymous_codon
PIK3CA	P2A	3_178921553_T/A	345	N/K	non_synonymous_codon
PIK3CA	P2B	3_178921553_T/A	345	N/K	non_synonymous_codon
RELN	M6A	7_103236925_T/C	1173	F/L	non_synonymous_codon
RELN	M6B	7_103236925_T/C	1173	F/L	non_synonymous_codon
RELN	P6A	7_103236925_T/C	1173	F/L	non_synonymous_codon
RELN	P6B	7_103236925_T/C	1173	F/L	non_synonymous_codon
RELN	P6C	7_103236925_T/C	1173	F/L	non_synonymous_codon
RYR2	P2A	1_237837494_C/G	2897	Q/E	non_synonymous_codon
RYR2	P2B	1_237837494_C/G	2897	Q/E	non_synonymous_codon
SPEN	M3A	1_16260941_G/T	2736	A/S	non_synonymous_codon
TP53	M2A	17_7577559_C/T	241	S/F	non_synonymous_codon
TP53	M6A	17_7577094_C/T	282	R/W	non_synonymous_codon
TP53	M6B	17_7577094_C/T	282	R/W	non_synonymous_codon
TP53	P2A	17_7577559_C/T	241	S/F	non_synonymous_codon

Gene Name	Sample	Variation	Protein position	Amino acids	Consequence
TP53	P2B	17_7577559_C/T	241	S/F	non_synonymous_codon
TP53	P6A	17_7577094_C/T	282	R/W	non_synonymous_codon
TP53	P6B	17_7577094_C/T	282	R/W	non_synonymous_codon
TP53	P6C	17_7577094_C/T	282	R/W	non_synonymous_codon
**FOM**					
**Gene Name**	**Sample**	**Variation**	**Protein_position**	**Amino acids**	**Consequence**
CSMD3	P3B	8_113649048_T/C	1238	V/A	non_synonymous_codon,splice_region_variant
FLG	M3A	1_152286341_G/A	341	E/K	non_synonymous_codon
HMCN1	M3A	1_185958647_G/T	1026	A/S	non_synonymous_codon
HMCN1	P3A	1_185958647_G/T	1026	A/S	non_synonymous_codon
HMCN1	P3B	1_185958647_G/T	1026	A/S	non_synonymous_codon
MLL3	M3A	7_151970877_C/T	309	P/S	non_synonymous_codon
PCLO	P3A	7_82595406_A/G	1172	K/R	non_synonymous_codon
SPEN	M3A	1_16260941_G/T	2736	A/S	non_synonymous_codon
**Larynx**					
**Gene Name**	**Sample**	**Variation**	**Protein_position**	**Amino acids**	**Consequence**
ADAMTS12	P7A	5_33641933_G/C	567	R/T	non_synonymous_codon
AR	P7A	X_66766583_C/T	532	S/F	non_synonymous_codon
ARID1A	P7A	1_27101099_C/T	1461	R/*	stop_gained
ATR	M1A	3_142215966_C/A	1876	S/Y	non_synonymous_codon
ATR	M1B	3_142215966_C/A	1876	S/Y	non_synonymous_codon
ATR	P1A	3_142215966_C/A	1876	S/Y	non_synonymous_codon
ATR	P1B	3_142215966_C/A	1876	S/Y	non_synonymous_codon
ATR	P7A	3_142280163_G/A	424	S/N	non_synonymous_codon
ATRX	P7B	X_76937555_G/C	1065	E/Q	non_synonymous_codon
BRCA2	P1A	13_32972329_C/G	3227	Q/E	non_synonymous_codon
BRCA2	P1B	13_32972329_C/G	3227	Q/E	non_synonymous_codon
CASP8	M1A	2_202137393_G/C	207	K/N	non_synonymous_codon
CASP8	M1A	2_202149922_G/A	455	E/K	non_synonymous_codon
CASP8	M1B	2_202137393_G/C	207	K/N	non_synonymous_codon
CASP8	M1B	2_202149922_G/A	455	E/K	non_synonymous_codon
CASP8	P1A	2_202137393_G/C	207	K/N	non_synonymous_codon
CASP8	P1A	2_202149922_G/A	455	E/K	non_synonymous_codon
CASP8	P1B	2_202137393_G/C	207	K/N	non_synonymous_codon
CASP8	P1B	2_202149922_G/A	455	E/K	non_synonymous_codon
CBL	P7A	11_119103173_C/G	71	Q/E	non_synonymous_codon
CBL	P7B	11_119103173_C/G	71	Q/E	non_synonymous_codon
CD19	P7A	16_28944345_T/C	157	Y/H	non_synonymous_codon
CDH10	P7B	5_24487962_C/A	726	T/N	non_synonymous_codon

Gene Name	Sample	Variation	Protein position	Amino acids	Consequence
CDH11	P7A	16_65038618_A/T	52	K/M	non_synonymous_codon
COL14A1	P7A	8_121243835_C/T	776	P/L	non_synonymous_codon
COL14A1	P7B	8_121209107_T/C	172	F/L	non_synonymous_codon
CREBBP	P7A	16_3788671_G/T	1428	R/L	non_synonymous_codon,splice_region_variant
CREBBP	P7B	16_3788671_G/T	1428	R/L	non_synonymous_codon,splice_region_variant
CSMD3	P7A	8_113299350_G/C	3092	E/Q	non_synonymous_codon
CSMD3	P7A	8_113529310_A/T	1570	Q/L	non_synonymous_codon
CSMD3	P7A	8_113562914_C/A	1517	A/E	non_synonymous_codon
CSMD3	P7A	8_113649225_T/G	1179	L/R	non_synonymous_codon
CSMD3	P7A	8_113668416_C/A	991	R/S	non_synonymous_codon
CSMD3	P7A	8_113933928_G/T	521	A/S	non_synonymous_codon
CSMD3	P7A	8_114186070_G/T	197	G/V	non_synonymous_codon
CSMD3	P7B	8_113326261_G/T	2524	V/L	non_synonymous_codon
CSMD3	P7B	8_113668416_C/A	991	R/S	non_synonymous_codon
DNMT3A	P7B	2_25505432_G/T	109	G/V	NMD_transcript_variant,non_synonymous_codon
DNMT3A	P7B	2_25505433_G/T	109	G/W	NMD_transcript_variant,non_synonymous_codon
FAM135B	P7A	8_139268942_A/T	120	S/C	non_synonymous_codon
FLG	M1A	1_152285836_C/A	509	S/Y	non_synonymous_codon
FLG	M1B	1_152285836_C/A	509	S/Y	non_synonymous_codon
FLG	P1A	1_152285836_C/A	509	S/Y	non_synonymous_codon
FLG	P1B	1_152285836_C/A	509	S/Y	non_synonymous_codon
FLT4	P7B	5_180046289_G/C	909	V/L	non_synonymous_codon
HMCN1	P7B	1_186017902_G/T	2170	G/C	non_synonymous_codon
HYDIN	P7A	16_71061711_A/G	946	M/V	non_synonymous_codon
HYDIN	P7B	16_71061711_A/G	946	M/V	non_synonymous_codon
JAK2	P7B	9_5126339_A/G	1062	M/V	non_synonymous_codon
KCNB2	P7B	8_73848509_C/A	307	R/S	non_synonymous_codon
KIT	P7A	4_55564653_C/T	181	R/W	non_synonymous_codon
KIT	P7B	4_55564653_C/T	181	R/W	non_synonymous_codon
LAMA1	P7B	18_6958546_G/T	2632	E/*	stop_gained
LPHN3	M1A	4_62936407_C/A	1397	S/R	non_synonymous_codon
LPHN3	M1B	4_62936407_C/A	1397	S/R	non_synonymous_codon
LPHN3	P1A	4_62936407_C/A	1397	S/R	non_synonymous_codon
LPHN3	P1B	4_62936407_C/A	1397	S/R	non_synonymous_codon
LPHN3	P7B	4_62758433_A/T	446	T/S	non_synonymous_codon
LRP1B	P7B	2_141215047_T/A	3267	Y/N	non_synonymous_codon
LRP1B	P7B	2_141458190_G/T	2143	G/V	non_synonymous_codon,splice_region_variant
LRP2	M1A	2_170048450_A/G	2975	D/G	non_synonymous_codon
LRP2	M1A	2_170058150_G/T	2814	E/*	stop_gained
LRP2	M1B	2_170048450_A/G	2975	D/G	non_synonymous_codon

Gene Name	Sample	Variation	Protein position	Amino acids	Consequence
LRP2	M1B	2_170058150_G/T	2814	E/*	stop_gained
LRP2	P1A	2_170048450_A/G	2975	D/G	non_synonymous_codon
LRP2	P1A	2_170058150_G/T	2814	E/*	stop_gained
LRP2	P1B	2_170048450_A/G	2975	D/G	non_synonymous_codon
LRP2	P1B	2_170058150_G/T	2814	E/*	stop_gained
LRP2	P7A	2_170034514_C/A	3398	H/N	non_synonymous_codon
MAP3K4	M1A	6_161470380_A/C	359	E/A	non_synonymous_codon
MAP3K4	M1B	6_161470380_A/C	359	E/A	non_synonymous_codon
MAP3K4	P1A	6_161470380_A/C	359	E/A	non_synonymous_codon
MAP3K4	P1B	6_161470380_A/C	359	E/A	non_synonymous_codon
MLL2	M1A	12_49444286_C/T	1029	Q/*	stop_gained
MLL2	M1B	12_49444286_C/T	1029	Q/*	stop_gained
MLL2	P1A	12_49444286_C/T	1029	Q/*	stop_gained
MLL2	P1B	12_49444286_C/T	1029	Q/*	stop_gained
MLL3	P1A	7_151970877_C/T	309	P/S	non_synonymous_codon
MLL3	P1A	7_151970951_G/A	284	R/Q	non_synonymous_codon,splice_region_variant
MLL3	P7A	7_151879451_A/T	1832	K/*	stop_gained
MLL3	P7B	7_151879451_A/T	1832	K/*	stop_gained
NAV3	P7B	12_78513547_C/A	1191	P/T	non_synonymous_codon
NCOR1	P7A	17_15961878_A/T	1973	I/F	non_synonymous_codon
NOTCH4	M1A	6_32170006_G/A	1201	G/E	non_synonymous_codon
NOTCH4	M1B	6_32170006_G/A	1201	G/E	non_synonymous_codon
NOTCH4	P1A	6_32170006_G/A	1201	G/E	non_synonymous_codon
NOTCH4	P1B	6_32170006_G/A	1201	G/E	non_synonymous_codon
PCDH15	P1A	10_55944926_G/T	477	D/Y	non_synonymous_codon
PCDH15	P1B	10_55944926_G/T	477	D/Y	non_synonymous_codon
PCDH15	P7B	10_55839154_G/T	683	L	synonymous_codon
PCLO	M1A	7_82580194_G/A	3168	W/*	stop_gained
PCLO	M1B	7_82580194_G/A	3168	W/*	stop_gained
PCLO	P1A	7_82580194_G/A	3168	W/*	stop_gained
PCLO	P1B	7_82580194_G/A	3168	W/*	stop_gained
PCLO	P7B	7_82585272_G/T	1597	R/L	non_synonymous_codon
PKHD1	P7B	6_51732736_A/T	2553	N/I	non_synonymous_codon
PKHD1L1	P7A	8_110530463_G/T	3919	M/I	non_synonymous_codon
PPP1R3A	P7A	7_113518083_G/T	1022	E/*	stop_gained
PPP1R3A	P7A	7_113518084_G/A	1021	M/I	non_synonymous_codon
RELN	P7B	7_103629751_G/T	18	G/V	non_synonymous_codon
RIMS2	P7A	8_104928716_G/T	471	E/*	stop_gained
RYR2	P7B	1_237982402_C/A	4834	P/T	non_synonymous_codon
SETD2	M1A	3_47165557_C/T	190	P/L	non_synonymous_codon

Gene Name	Sample	Variation	Protein position	Amino acids	Consequence
SETD2	M1B	3_47165557_C/T	190	P/L	non_synonymous_codon
SETD2	P1A	3_47165557_C/T	190	P/L	non_synonymous_codon
SETD2	P1B	3_47165557_C/T	190	P/L	non_synonymous_codon
SPEN	P1B	1_16242703_A/C	442	I/L	non_synonymous_codon
SYNE1	M1A	6_152529268_C/G	7555	R/G	non_synonymous_codon
SYNE1	M1B	6_152529268_C/G	7555	R/G	non_synonymous_codon
SYNE1	P7A	6_152720907_G/C	2361	E/Q	non_synonymous_codon
SYNE1	P7B	6_152720907_G/C	2361	E/Q	non_synonymous_codon
TOP1	P7A	20_39742763_A/G	536	R/G	non_synonymous_codon
TOP1	P7A	20_39746814_G/A	610	E/K	non_synonymous_codon
TOP1	P7B	20_39742763_A/G	536	R/G	non_synonymous_codon
TP53	M1A	17_7577058_G/T	294	E/*	stop_gained
TP53	M1B	17_7577058_G/T	294	E/*	stop_gained
TP53	P1A	17_7577058_G/T	294	E/*	stop_gained
TP53	P1B	17_7577058_G/T	294	E/*	stop_gained
TP53	P7A	17_7577529_T/A	251	I/N	non_synonymous_codon
TP53	P7A	17_7577574_A/G	236	Y/C	non_synonymous_codon
TP53	P7B	17_7577529_T/A	251	I/N	non_synonymous_codon
USH2A	P7A	1_215987204_C/G	3205	R/G	non_synonymous_codon
USH2A	P7B	1_215987204_C/G	3205	R/G	non_synonymous_codon
USH2A	P7B	1_216219931_C/A	2056	P/Q	non_synonymous_codon

Supplementary Table 4: CNVs in oral tongue, FOM and laryngeal tumors..

**Oral Tongue**			
**Gene Name**	**Sample**	**CNS**	**Copy Number (CN)**
ABL1	M5B	AMP	2.5
ABL1	P5A	AMP	2.5
ABL1	P5C	AMP	2.6
AKAP3	P6A	AMP	2.6
BAP1	P6C	DEL	1.5
BRCA2	P6C	DEL	1.5
CDKN2A	M5B	AMP	2.5
CDKN2A	P5A	AMP	2.5
CDKN2A	P5C	AMP	2.6
CNS	P4B	H.AMP	7.2
COL14A1	M5A	AMP	2.6
COL14A1	M5B	AMP	3
COL14A1	P5A	AMP	3.2
COL14A1	P5B	AMP	3.1
COL14A1	P5C	AMP	3.2
COL14A1	P6A	AMP	2.6
COL14A1	P6C	AMP	2.6
CRIPAK	M2A	AMP	2.5
CSMD1	P6A	DEL	1.5
CSMD1	P6C	DEL	1.4
CSMD3	M5A	AMP	2.6
CSMD3	M5B	AMP	3
CSMD3	P5A	AMP	3.2
CSMD3	P5B	AMP	3.1
CSMD3	P5C	AMP	3.2
CSMD3	P6A	AMP	2.6
CSMD3	P6C	AMP	2.6
CTNNB1	P6C	DEL	1.5
EGFR	P4A	H.AMP	6.6
EPHA3	P6C	DEL	1.5
FAM135B	M5A	AMP	2.6
FAM135B	M5B	AMP	3
FAM135B	P5A	AMP	3.2
FAM135B	P5B	AMP	3.1
FAM135B	P5C	AMP	3.2
FAM135B	P6A	AMP	2.6
FAM135B	P6C	AMP	2.6

FGFR1	P6A	DEL	1.5
**Gene Name**	**Sample**	**CNS**	**Copy Number (CN)**
FGFR1	P6C	DEL	1.4
FGFR3	M2A	AMP	2.5
FLT1	P6C	DEL	1.5
FLT3	P6C	DEL	1.5
FLT4	M6A	NORM,AMP	2,2.8,2
GATA3	P6A	DEL	1.4
GATA3	P6C	DEL	1.5
GNA11	P6A	DEL	1.4
GNAQ	M5B	AMP	2.5
GNAQ	P5A	AMP	2.5
GNAQ	P5C	AMP	2.6
HNF1B	P6C	NORM,AMP	2,2.6
JAK2	M5B	AMP	2.5
JAK2	P5A	AMP	2.5
JAK2	P5C	AMP	2.6
KCNB2	M5A	AMP	2.6
KCNB2	M5B	AMP	3
KCNB2	P5A	AMP	3.2
KCNB2	P5B	AMP	3.1
KCNB2	P5C	AMP	3.2
KCNB2	P6A	AMP	2.6
KCNB2	P6C	AMP	2.6
KRAS	P6A	AMP	2.6
MDN1	P4A	AMP	2.5
MITF	P6C	DEL	1.5
MLH1	P6C	DEL	1.5
MYD88	P6C	DEL	1.5
NOTCH1	M5B	AMP	2.5
NOTCH1	P5A	AMP	2.5
NOTCH1	P5C	AMP	2.6
PAX5	M5B	AMP	2.5
PAX5	P5A	AMP	2.5
PAX5	P5C	AMP	2.6
PBRM1	P6C	DEL	1.5
PKHD1	M6A	AMP,NORM	2.8,1.9
PKHD1	M6B	AMP,NORM	3.6,1.9
PKHD1	P6A	AMP,NORM	3,1.9
PKHD1	P6B	AMP,NORM	3.2,1.9

PKHD1	P6C	AMP,NORM	3.4,1.9
**Gene Name**	**Sample**	**CNS**	**Copy Number (CN)**
PKHD1L1	M5A	AMP	2.6
PKHD1L1	M5B	AMP	3
PKHD1L1	P5A	AMP	3.2
PKHD1L1	P5B	AMP	3.1
PKHD1L1	P5C	AMP	3.2
PKHD1L1	P6A	AMP	2.6
PKHD1L1	P6C	AMP	2.6
PPP2R4	M5B	AMP	2.5
PPP2R4	P5A	AMP	2.5
PPP2R4	P5C	AMP	2.6
PTCH1	M5B	AMP	2.5
PTCH1	P5A	AMP	2.5
PTCH1	P5C	AMP	2.6
PTK2	M5A	AMP	2.6
PTK2	M5B	AMP	3
PTK2	P5A	AMP	3.2
PTK2	P5B	AMP	3.1
PTK2	P5C	AMP	3.2
PTK2	P6A	AMP	2.6
PTK2	P6C	AMP	2.6
RAF1	P6C	DEL	1.5
RIMS2	M5A	AMP	2.6
RIMS2	M5B	AMP	3
RIMS2	P5A	AMP	3.2
RIMS2	P5B	AMP	3.1
RIMS2	P5C	AMP	3.2
RIMS2	P6A	AMP	2.6
RIMS2	P6C	AMP	2.6
RNF213	P6C	AMP	2.6
RUNX1T1	M5A	AMP	2.6
RUNX1T1	M5B	AMP	3
RUNX1T1	P5A	AMP	3.2
RUNX1T1	P5B	AMP	3.1
RUNX1T1	P5C	AMP	3.2
RUNX1T1	P6A	AMP	2.6
RUNX1T1	P6C	AMP	2.6
SETD2	P6C	DEL	1.5
SMAD4	M6B	DEL	1.3
SMAD4	P6A	DEL	1.3

SMAD4	P6B	DEL	1.4
**Gene Name**	**Sample**	**CNS**	**Copy Number (CN)**
SMAD4	P6C	DEL	1.1
SMARCA4	P6A	DEL,NORM	1.4,1.8
STK11	M5B	DEL	1.3
STK11	P5A	DEL	1.2
STK11	P5B	DEL	1.1
STK11	P5C	DEL	1.2
STK11	P6A	DEL	1.4
SYK	M5B	AMP	2.5
SYK	P5A	AMP	2.5
SYK	P5C	AMP	2.6
TGFBR2	P6C	DEL	1.5
TNFAIP3	P4A	AMP	2.5
TSC1	M5B	AMP	2.5
TSC1	P5A	AMP	2.5
TSC1	P5C	AMP	2.6
VHL	P6C	DEL	1.5
WHSC1	M2A	AMP,NORM	2.5,2
**FOM**			
**Gene Name**	**Sample**	**CNS**	**Copy Number (CN)**
AKT1	M3A	AMP	2.7
APC	M3A	DEL	1.4
ADAMTS12	P3A	AMP,NORM	2.5,1.9,2.5
ADAMTS12	P3B	AMP	2.6
AKT1	P3A	AMP	2.9
AKT1	P3B	AMP	2.8
ALK	P3A	AMP	2.7
APC	P3A	DEL	1.4
APC	P3B	DEL	1.4
AR	P3A	AMP,NORM	2.8,1.7
AR	P3B	AMP	2.6
ARAF	P3A	AMP	2.8
ARAF	P3B	AMP	2.6
ATR	P3A	NORM,H.DEL	1.8,0.9,1.7
AURKB	P3A	AMP	2.5
BAI3	P3A	DEL	1.5
BAP1	M3A	AMP,NORM	2.7,1.8,2.6
BAP1	P3B	DEL	1.3
BRAF	P3A	NORM,AMP	1.7,2.7

BRAF	P3B	NORM,AMP	1.8,2.6
**Gene Name**	**Sample**	**CNS**	**Copy Number (CN)**
BRCA2	M3A	DEL	1.5
BRCA2	P3A	DEL	1.5
CARD11	P3A	AMP	2.7
CBL	M3A	NORM,DEL	2.3,1.4
CBL	P3B	NORM,DEL	2.1,1.2
CD19	P3A	AMP	2.8
CDH10	P3B	AMP	2.6
CDKN2A	M3A	AMP	3
CDKN2A	P3A	AMP	3
CDKN2A	P3B	DEL	1.1
CHEK1	M3A	DEL	1.4
CHEK1	P3B	DEL	1.2
COL14A1	P3A	DEL,H.DEL,NORM	1.5,0.7,1.5,2.1
CPAMD8	M3A	AMP	2.6
CPAMD8	P3A	AMP	2.9
CPAMD8	P3B	AMP,H.AMP	2.9,4.3,2.7
CREBBP	M3A	AMP,NORM	2.8,2.1
CREBBP	P3A	AMP	2.8
CREBBP	P3B	AMP,NORM	3,2.3
CRIPAK	M3A	AMP	3.5
CRIPAK	P3A	AMP	3.4
CRIPAK	P3B	AMP	3.3
CSF1R	P3B	DEL	1.4
CSMD1	P3A	DEL,NORM	1.5,2.2
CSMD3	P3A	DEL	1.5
CTNNB1	P3B	DEL	1.3
CYP2C19	P3B	DEL	1.5
DAXX	P3A	AMP	2.7
DDR1	P3A	AMP	2.7
DNMT3A	P3A	AMP	2.7
EGFR	P3A	AMP	2.7
ELN	P3A	AMP	2.7
EP300	M3A	NORM,AMP	2,2.8
EP300	P3A	NORM,AMP	2.2,3
EP300	P3B	NORM,AMP	2.2,2.8
EPHA3	M3A	DEL	1.5
EPHA3	P3A	NORM,DEL	1.6,1,1.8

EPHA3	P3B	DEL	1.3
**Gene Name**	**Sample**	**CNS**	**Copy Number (CN)**
ERBB2	M3A	AMP	2.5
ERBB2	P3A	AMP	2.8
ERCC3	P3A	NORM,AMP	1.9,3.3
ERCC4	P3A	AMP,NORM	2.8,2
ERCC5	P3A	AMP,NORM	2.9,1.8
FAM123B	P3A	AMP	2.8
FAM123B	P3B	AMP	2.6
FGFR2	P3B	DEL	1.5
FGFR3	M3A	AMP	3.5
FGFR3	P3A	AMP	3.4
FGFR3	P3B	AMP	3.3
FGFR4	M3A	DEL,NORM	1.5,2.1
FGFR4	P3B	DEL	1.4
FLT1	M3A	DEL	1.5
FLT1	P3A	DEL	1.5
FLT3	M3A	DEL	1.5
FLT3	P3A	DEL	1.5
FLT4	P3B	DEL	1.4
GABRA6	M3A	DEL	1.5
GABRA6	P3A	DEL,NORM	1.2,1.5
GABRA6	P3B	DEL	1.4
GABRB3	P3A	AMP	2.5
GATA1	P3A	AMP	2.8
GATA1	P3B	AMP	2.6
GATA3	P3A	AMP	2.7
GNA11	M3A	AMP	2.6
GNA11	P3A	AMP	2.9
GNA11	P3B	AMP	2.9
HEATR7B2	P3A	AMP,NORM	2.5,1.9
HEATR7B2	P3B	AMP	2.6
HMCN1	P3A	NORM,DEL,H.DEL	1.8,1.3,0.7,1.4,1.8,1.3,1.8
HNF1A	M3A	AMP	2.6
HNF1A	P3A	AMP	2.8
HNF1B	M3A	AMP	2.5
HNF1B	P3A	AMP	2.8
HRAS	P3A	AMP	2.8
IDH2	P3A	AMP	2.5

IGF1R	P3A	AMP	2.5
**Gene Name**	**Sample**	**CNS**	**Copy Number (CN)**
IKZF1	P3A	NORM,AMP	1.7,2.7
ITGA4	M3A	AMP,NORM	3.6,1.7
ITGA4	P3A	AMP,DEL	3.5,1.3
JAK2	M3A	DEL	1.4
JAK2	P3A	DEL	1.2
JAK2	P3B	DEL	1.1
JAK3	M3A	AMP	2.6
JAK3	P3A	AMP	2.9
JAK3	P3B	AMP	2.7
LRP1B	P3A	H.DEL,DEL,NORM	0.4,1.1,0.6,1.2,1.7
LRP2	M3A	NORM,AMP	1.8,3.6
LRP2	P3A	NORM,AMP	1.7,3.5
MAP2K1	P3A	AMP	2.5
MAP3K1	M3A	NORM,DEL	2.4,1.4
MAP3K1	P3A	NORM,DEL	1.9,1.4
MAP3K1	P3B	AMP,DEL	2.6,1.4
MEN1	P3A	AMP	2.9
MITF	M3A	DEL	1.5
MITF	P3B	DEL	1.3
MLH1	P3B	DEL	1.3
MYD88	P3B	DEL	1.3
NAV3	P3A	NORM,DEL	1.7,1.1
NF1	M3A	NORM,AMP	1.9,2.5
NF1	P3A	NORM,AMP	1.9,2.8
NFKB2	M3A	NORM,AMP	2.1,2.8,1.9
NFKB2	P3A	AMP,NORM	2.6,3.3,2.3
NFKB2	P3B	DEL	1.5
NOTCH1	M3A	AMP	3
NOTCH1	P3A	AMP	3.1
NOTCH3	M3A	AMP	2.6
NOTCH3	P3A	AMP	2.9
NOTCH3	P3B	AMP	2.9
NOTCH4	P3A	AMP	2.7
NPM1	M3A	DEL	1.5
NPM1	P3B	DEL	1.4
NSD1	P3B	DEL	1.4
PALB2	P3A	NORM,AMP	2,2.8

PAX5	M3A	AMP,NORM	3,2.3
**Gene Name**	**Sample**	**CNS**	**Copy Number (CN)**
PAX5	P3A	AMP	3
PAX5	P3B	AMP	3.7
PBRM1	M3A	DEL	1.5
PBRM1	P3B	DEL	1.3
PCDH15	P3B	DEL	1.5
PDGFRB	P3B	DEL	1.4
PIK3R1	M3A	DEL	1.4
PIK3R1	P3A	DEL	1.4
PIK3R1	P3B	DEL	1.4
PKHD1	P3A	AMP,NORM,DEL	2.7,1.5,2.2,1.7,2.3,1.5
PKHD1L1	P3A	DEL	1.5
PPP2R4	M3A	AMP,NORM	3.4,2.2
PRSS1	P3A	AMP	2.7
PRSS1	P3B	AMP	2.6
PTCH1	M3A	NORM,AMP	2.3,3.4
PTEN	P3B	DEL	1.5
RAD51	P3A	AMP	2.5
RAF1	P3B	DEL	1.3
RB1	M3A	DEL	1.5
RB1	P3A	DEL	1.5
RET	P3A	AMP	2.7
RET	P3B	NORM,DEL	2.3,1.5
RIMS2	P3A	NORM,DEL	2.2,1.5
RNF213	P3A	AMP	2.6
SETD2	M3A	NORM,AMP	1.5,2.7
SETD2	P3B	DEL	1.3
SMAD4	M3A	DEL	1.3
SMAD4	P3B	DEL	1.2
SMARCA4	M3A	AMP	2.6
SMARCA4	P3A	AMP	2.9
SMARCA4	P3B	AMP	2.9
SMARCB1	M3A	AMP	2.6
SMARCB1	P3A	AMP	2.8
SMO	P3A	AMP	2.8
SMO	P3B	AMP	2.6
STK11	M3A	AMP	2.6
STK11	P3A	AMP	2.9

STK11	P3B	AMP	2.9
**Gene Name**	**Sample**	**CNS**	**Copy Number (CN)**
SYNE2	P3A	NORM,AMP	2,2.6
TBC1D4	M3A	DEL,NORM	1.5,2.4
TBC1D4	P3A	DEL,AMP	1.5,2.9
TGFBR2	P3B	DEL	1.3
TP53	P3A	AMP	2.5
TSC2	M3A	NORM,AMP	2.1,2.8
TSC2	P3A	AMP	2.8
TSC2	P3B	NORM,AMP	2.3,3
TSHR	M3A	NORM,AMP	2,2.7
TSHR	P3A	AMP,NORM	2.6,1.7,2.9
TSHR	P3B	NORM,AMP	2.1,2.8
VHL	P3B	DEL	1.3
WT1	P3A	NORM,AMP	2.1,2.9
ZNF536	M3A	AMP,NORM	2.6,1.8
ZNF536	P3A	AMP,NORM	2.9,2.2
**Larynx**			
**Gene Name**	**Sample**	**CNS**	**Copy Number (CN)**
ABL1	P1B	AMP	2.6
ACVR1B	M1B	AMP	3.1
ACVR1B	P1B	AMP	3
ADAMTS12	P7B	AMP	2.7
AKAP3	M1A	H.AMP	5.6
AKAP3	P1A	H.AMP,NORM	5.6,2.1
APC	P1B	DEL,AMP	1,2.7
AR	M1B	AMP	2.5
AR	P1B	AMP	2.7
AR	P7A	DEL	1.5
AR	P7B	DEL	1.4
ARAF	M1B	AMP	2.5
ARAF	P1B	AMP	2.7
ARAF	P7A	DEL	1.5
ARAF	P7B	DEL	1.1,1.4
ATM	M1A	DEL	1.4
ATM	M1B	H.DEL,DEL	0.7,1.1
ATM	P1A	DEL	1.4
ATM	P1B	H.DEL,NORM	0.6,1.5
ATM	P7A	DEL	1.5
ATR	M1B	DEL	1.3

ATR	P1B	DEL	1.3
**Gene Name**	**Sample**	**CNS**	**Copy Number (CN)**
ATRX	M1B	AMP,H.DEL	2.5,1
ATRX	P1B	AMP,H.DEL	2.7,0.8
ATRX	P7A	DEL	1.5
ATRX	P7B	DEL	1.4
BAI3	M1B	NORM,H.DEL	2.1,1,1.9
BAI3	P1B	NORM,H.DEL	2.3,1,2
BAP1	M1A	DEL	1.4
BAP1	M1B	DEL	1.3
BAP1	P1A	DEL	1.4
BAP1	P1B	DEL	1.3
BRAF	M1A	DEL	1.5
BRAF	M1B	DEL	1.2
BRAF	P1A	DEL	1.4
BRCA1	M1A	DEL	1.5
BRCA1	M1B	DEL	1.2
BRCA1	P1A	DEL	1.4
BRCA1	P1B	H.DEL	0.8
BRCA2	M1A	AMP,H.AMP	4,4.9
BRCA2	M1B	AMP	2.8
BRCA2	P1A	NORM,AMP	1.7,2.9
BRCA2	P1B	NORM,H.DEL,DEL	2.1,0.5,1.4
BRCA2	P7B	DEL	1.5
CARD11	P1A	AMP	2.7
CARD11	P1B	AMP	2.7
CARD11	P7B	AMP	3.2
CASP8	M1B	DEL	1.3
CASP8	P7B	DEL	1.4
CBL	M1A	DEL	1.4
CBL	M1B	DEL	1.1
CBL	P1A	DEL	1.4
CBL	P7A	DEL	1.5
CD19	P1B	AMP	2.6
CDH10	M1A	H.AMP	4
CDH10	M1B	AMP	3.1
CDH10	P1A	AMP	2.6
CDH10	P7B	AMP	2.7
CDK4	M1A	AMP	2.6
CDK4	M1B	AMP	3.1
CDK4	P1A	AMP	2.7

CDK4	P1B	AMP	3
**Gene Name**	**Sample**	**CNS**	**Copy Number (CN)**
CDKN2A	P1B	H.DEL,AMP	0.8,2.6
CEBPA	P1B	AMP	2.6
CHEK1	M1A	DEL	1.4
CHEK1	M1B	DEL	1.1
CHEK1	P1A	DEL	1.4
CHEK1	P1B	NORM,H.DEL	1.5,0.7
CHEK1	P7A	DEL	1.5
CHEK2	P7A	AMP	2.8
CHEK2	P7B	AMP	2.8
COL14A1	P7B	AMP	2.5
CPAMD8	P1B	AMP	2.6
CPAMD8	P7A	AMP	2.9
CREBBP	P1B	AMP	2.6
CRIPAK	M1A	AMP	3
CRIPAK	M1B	AMP	3.8
CRIPAK	P1A	AMP	3.2
CRIPAK	P1B	H.AMP	4.5
CSF1R	P1B	AMP	2.7
CSMD1	M1A	DEL	1.4
CSMD1	M1B	DEL,NORM	1.3,2.5
CSMD1	P1A	DEL	1.4
CSMD1	P1B	DEL	1.4
CSMD1	P7B	NORM,H.DEL,AMP	2.1,1,2.5
CSMD3	M1B	DEL,NORM	1.3,2.1
CSMD3	P1B	H.DEL,NORM	0.9,1.8
CSMD3	P7B	AMP	2.5
CTNNB1	M1A	DEL	1.4
CTNNB1	M1B	DEL	1.3
CTNNB1	P1A	DEL	1.4
CTNNB1	P1B	DEL	1.3
CYLD	M1B	NORM,DEL	2.3,1.1,2.1
CYLD	P1B	AMP,H.DEL,NORM	2.6,1,2.3
CYP2C19	P1B	H.DEL,NORM	0.8,2.1
DNMT3A	P7B	NORM,AMP	2.4,3
EGFR	P1B	AMP	2.7
EGFR	P7B	AMP	3.2
ELN	P1B	AMP	2.7
ELN	P7B	AMP,NORM	3.2,2.2
EML4	M1B	DEL	1.2

EML4	P1B	H.DEL	1
**Gene Name**	**Sample**	**CNS**	**Copy Number (CN)**
EP300	M1A	DEL	1.5
EP300	P1A	DEL	1.4
EP300	P7A	AMP	2.8
EP300	P7B	AMP,NORM	2.8,2.1,2.7
EPHA3	M1A	DEL	1.4
EPHA3	M1B	DEL	1.3
EPHA3	P1A	DEL	1.4
EPHA3	P1B	DEL	1.3
ERBB2	M1A	DEL	1.5
ERBB2	M1B	DEL	1.2
ERBB2	P1A	DEL	1.4
ERBB3	M1A	AMP	2.6
ERBB3	M1B	AMP	3.1
ERBB3	P1A	AMP	2.7
ERBB3	P1B	AMP	3
ERCC3	P1B	AMP	2.6
ERCC4	P1B	AMP,NORM	2.6,1.5
ERCC5	M1A	DEL	1.4
ERCC5	M1B	DEL	1.1
ERCC5	P1A	DEL	1.4
ERCC5	P1B	DEL	1
ERCC5	P7B	DEL	1.5
ETV5	M1B	AMP	2.6
ETV5	P1B	DEL	1.3
EZH2	M1A	DEL	1.5
EZH2	M1B	DEL	1.2
EZH2	P1A	DEL	1.4
FAM123B	M1B	AMP	2.5
FAM123B	P1B	AMP	2.7
FAM123B	P7A	DEL	1.5
FAM123B	P7B	DEL	1.4
FAM135B	P7B	AMP	2.5
FAT3	M1A	DEL	1.4
FAT3	P1A	AMP	2.6
FBXW7	M1B	DEL	1.3
FBXW7	P1B	DEL	1.2
FGFR1	P1B	AMP	2.7
FGFR1	P7B	AMP	2.5
FGFR3	M1A	AMP,NORM	3,1.9

FGFR3	M1B	AMP	3.8
**Gene Name**	**Sample**	**CNS**	**Copy Number (CN)**
FGFR3	P1A	AMP,NORM	3.2,2
FGFR3	P1B	H.AMP	4.5
FGFR4	M1A	AMP	2.6
FGFR4	P1A	AMP	2.7
FGFR4	P1B	AMP	3.2
FLT1	M1A	NORM,AMP	1.9,4
FLT1	M1B	NORM,AMP	1.8,2.8
FLT1	P1A	NORM,AMP	2.2,3.1,1.7
FLT1	P7B	DEL	1.5
FLT3	P7B	DEL	1.5
FLT4	M1A	AMP	2.6
FLT4	P1A	AMP	2.7
FLT4	P1B	AMP	3.2
FOXL2	M1B	DEL	1.3
FOXL2	P1A	DEL	1.4
FOXL2	P1B	DEL	1.3
GABRA6	P1B	AMP,DEL	2.7,1.4
GABRB3	M1A	DEL	1.4
GABRB3	M1B	DEL	1.4
GABRB3	P1A	DEL	1.4
GABRB3	P1B	DEL	1.5
GABRB3	P7B	DEL	1.4
GATA1	M1B	AMP	2.5
GATA1	P1B	AMP	2.7
GATA1	P7A	DEL	1.5
GATA1	P7B	DEL	1.4
GATA3	M1A	DEL	1.5
GNA11	P1A	DEL	1.3
GNA11	P1B	AMP	2.6
GNAQ	P1B	AMP	2.6
HDAC9	P1B	AMP	2.7
HDAC9	P7B	AMP	3.2
HEATR7B2	P1B	NORM,DEL	2.1,1
HEATR7B2	P7B	AMP	2.7
HGF	M1B	NORM,DEL	2.3,1.1
HGF	P1B	AMP,NORM	2.7,1.5
HMCN1	P1B	NORM,DEL	2.3,1.3
HNF1A	M1A	AMP	2.6
HNF1A	M1B	AMP	2.9

HNF1A	P1A	AMP	3.5
**Gene Name**	**Sample**	**CNS**	**Copy Number (CN)**
HNF1A	P1B	AMP	3.8
HNF1B	M1A	DEL	1.5
HNF1B	M1B	DEL	1.2
HNF1B	P1A	DEL	1.4
HRAS	M1A	DEL	1.4
HRAS	P1A	DEL	1.4
IDH1	M1B	DEL	1.3
IDH1	P7B	DEL	1.4
IDH2	P1B	AMP	2.7
IGF1R	P1B	AMP	2.7
IKZF1	P1B	AMP	2.7
IKZF1	P7B	AMP	3.2
IRS1	M1B	DEL	1.3
IRS1	P1B	DEL	1
IRS1	P7B	DEL	1.4
ITGA4	M1B	NORM,DEL	2.1,1.3
ITGA4	P1B	NORM,H.DEL	2.1,0.9
JAK2	M1B	H.DEL,NORM	0.9,2.4
JAK2	P1B	H.DEL	0.8
JAK3	P1B	AMP	2.6
JAK3	P7A	AMP	2.9
KCNB2	P1B	AMP	2.7
KCNB2	P7B	AMP	2.5
KDM6A	M1B	DEL	1.3
KDM6A	P1B	DEL	1.2
KDM6A	P7A	DEL	1.5
KDM6A	P7B	DEL	1.1
KDR	M1B	NORM,DEL	2.1,1.3
KDR	P1B	NORM,DEL	2.2,1.2
LAMA1	P1A	NORM,DEL	2,1.4
LAMA1	P7A	AMP	2.8
LPHN3	M1B	DEL	1.3
LPHN3	P1B	DEL	1.2
LRP1	M1A	AMP	2.6
LRP1	M1B	AMP	3.1
LRP1	P1A	AMP	2.7
LRP1	P1B	AMP	3
LRP1B	M1A	NORM,DEL	1.9,1.4,1.9
LRP1B	M1B	DEL,NORM	1.1,2.1

LRP1B	P1A	NORM,DEL	2,1.4,2
**Gene Name**	**Sample**	**CNS**	**Copy Number (CN)**
LRP1B	P1B	DEL,NORM	1,2.1
LRP1B	P7B	NORM,DEL,H.DEL	2.1,1.3,1.7,1,2.1
MAP2K1	M1B	DEL,NORM	1.4,2.4
MAP2K1	P1B	DEL,AMP	1.5,2.7
MAP3K1	P1A	DEL	1.4
MAP3K1	P1B	DEL	1
MECOM	M1B	DEL	1.3
MECOM	P1B	DEL	1.3
MEN1	M1B	AMP	2.7
MEN1	P1A	DEL,NORM	1.4,2
MET	M1A	DEL	1.5
MET	M1B	DEL	1.2
MET	P1A	DEL	1.4
MITF	M1A	DEL	1.4
MITF	M1B	DEL	1.3
MITF	P1A	DEL	1.4
MITF	P1B	DEL	1.3
MLH1	M1A	DEL	1.4
MLH1	M1B	DEL	1.3
MLH1	P1A	DEL	1.4
MLH1	P1B	DEL	1.3
MLL2	M1B	AMP	3.1
MLL2	P1B	AMP	3
MLL3	M1A	DEL	1.5
MLL3	M1B	DEL	1.2
MLL3	P1A	DEL	1.4
MLL3	P1B	NORM,H.DEL	1.5,0.8
MSH2	M1B	DEL	1.2
MSH2	P1B	H.DEL	1
MSH6	P1B	NORM,DEL	2.3,1.2
MTOR	M1A	AMP	2.6
MTOR	P1A	AMP	2.7
MYD88	M1A	DEL	1.4
MYD88	M1B	DEL	1.3
MYD88	P1A	DEL	1.4
MYD88	P1B	DEL	1.3
NAV3	M1A	AMP	2.6
NAV3	M1B	AMP,NORM	3.1,1.6,2.9
NAV3	P1A	AMP	2.7

NAV3	P1B	AMP,DEL	3,1.4,3.8
**Gene Name**	**Sample**	**CNS**	**Copy Number (CN)**
NCOR1	M1B	NORM,DEL	1.9,1.2
NCOR1	P1B	NORM,DEL	2,1
NF1	M1A	NORM,DEL	2,1.5
NF1	M1B	DEL	1.2
NF1	P1A	DEL	1.4
NF1	P1B	DEL,NORM	1,1.9
NF2	P7A	AMP	2.8
NF2	P7B	AMP	2.8
NOTCH1	P1B	AMP	2.6
NOTCH2	M1A	AMP	3.1
NOTCH2	M1B	AMP	3.7
NOTCH3	P1B	AMP	2.6
NOTCH3	P7A	AMP	2.9
NPM1	P1A	AMP	2.7
NPM1	P1B	DEL	1.4
NSD1	M1A	AMP	2.6
NSD1	P1A	AMP	2.7
NSD1	P1B	AMP	3.2
PALB2	P1B	NORM,AMP	1.5,2.6
PAX5	P1B	AMP	2.6
PBRM1	M1A	DEL	1.4
PBRM1	M1B	DEL	1.3
PBRM1	P1A	DEL	1.4
PBRM1	P1B	DEL	1.3
PCDH15	M1B	NORM,H.DEL	2.3,1
PCDH15	P1B	NORM,H.DEL	2.1,0.8
PCLO	M1B	DEL,NORM	1.1,1.9
PDGFRB	P1B	AMP	2.7
PIK3CA	M1B	DEL,AMP	1.3,2.6
PIK3CA	P1B	DEL	1.3
PIK3CG	P1A	AMP	2.6
PIK3R1	P1A	DEL	1.4
PIK3R1	P1B	DEL	1
PIKFYVE	M1B	DEL	1.3
PIKFYVE	P1B	NORM,DEL	1.8,1
PIKFYVE	P7B	DEL	1.4
PKHD1L1	M1B	DEL	1.3
PKHD1L1	P1B	DEL,H.DEL	1.4,0.9
PKHD1L1	P7B	AMP	2.5

PPP1R3A	M1B	NORM,H.DEL,DEL	1.9,0.6,1.2
**Gene Name**	**Sample**	**CNS**	**Copy Number (CN)**
PPP1R3A	P1A	AMP	2.6
PPP2R1A	P1B	AMP	2.6
PPP2R4	P1B	AMP	2.6
PRDM1	P1A	NORM,DEL	2,1.4
PRSS1	M1A	DEL	1.5
PRSS1	M1B	DEL	1.2
PRSS1	P1A	DEL	1.4
PTCH1	P1B	AMP	2.6
PTEN	M1B	H.DEL,NORM	1,2.3
PTEN	P1B	H.DEL	0.8
PTK2	P1A	AMP	2.6
PTK2	P1B	AMP,DEL	3.1,1.3
PTK2	P7B	AMP	2.5
PTPN11	M1A	AMP	2.6
PTPN11	M1B	AMP	2.9
PTPN11	P1A	AMP	2.7,3.5
PTPN11	P1B	AMP	3.8
RAD51	M1A	DEL	1.4
RAD51	M1B	DEL	1.4
RAD51	P1A	DEL	1.4
RAD51	P1B	DEL	1.5
RAD51	P7B	DEL	1.4
RAF1	M1A	DEL	1.4
RAF1	M1B	DEL	1.3
RAF1	P1A	DEL	1.4
RAF1	P1B	DEL	1.3
RB1	M1A	H.AMP,DEL	4.9,1.4
RB1	M1B	AMP,H.DEL,DEL	2.8,0.4,1.1
RB1	P1A	DEL	1.4
RB1	P1B	DEL,H.DEL	1.4,0.4,1
RB1	P7B	DEL	1.5
RIMS2	M1B	NORM,DEL	2.5,1.3
RIMS2	P1B	AMP,DEL	2.7,1.4
RIMS2	P7B	AMP	2.5
RNF213	P1B	AMP	2.6
RUNX1T1	P1B	AMP	2.7
RUNX1T1	P7B	AMP	2.5
RYR2	P1B	DEL	1.3
SETD2	M1A	DEL	1.4

SETD2	M1B	DEL	1.3
**Gene Name**	**Sample**	**CNS**	**Copy Number (CN)**
SETD2	P1A	DEL	1.4
SETD2	P1B	DEL	1.3
SMAD4	M1A	DEL	1.4
SMAD4	M1B	DEL	1
SMAD4	P1A	DEL	1.4
SMAD4	P1B	DEL	1.1
SMAD4	P7B	DEL	1.4
SMARCA4	P1B	AMP	2.6
SMARCB1	P7A	AMP	2.8
SMARCB1	P7B	AMP	2.8
SMO	M1A	DEL	1.5
SMO	M1B	DEL	1.2
SMO	P1A	DEL	1.4
SOS1	M1B	NORM,DEL	2.3,1.2
SOS1	P1B	NORM,H.DEL	2.5,1
SPEN	M1A	AMP,NORM	2.6,2
SPOP	P1B	AMP	2.6
STK11	P1A	DEL	1.3
STK11	P1B	AMP	2.6
SYK	P1B	AMP	2.6
SYNE1	M1B	NORM,H.DEL	1.9,0.5,1.7
SYNE1	P1B	NORM,H.DEL	2,0.5,1.8
SYNE2	M1B	DEL,NORM	1.4,2
SYNE2	P1B	DEL,NORM	1.1,2
TBC1D4	M1A	DEL	1.4
TBC1D4	M1B	DEL	1.1
TBC1D4	P1A	DEL	1.4
TBC1D4	P1B	DEL	1
TBC1D4	P7B	DEL	1.5
TET2	M1B	DEL	1.3
TET2	P1B	DEL	1.2
TGFB1	P1B	AMP	2.6
TGFBR2	M1A	DEL	1.4
TGFBR2	M1B	DEL	1.3
TGFBR2	P1A	DEL	1.4
TGFBR2	P1B	DEL	1.3
TOP2A	M1A	DEL	1.5
TOP2A	M1B	DEL	1.2
TOP2A	P1A	DEL	1.4

TOP2A	P1B	NORM,H.DEL	1.9,0.8
**Gene Name**	**Sample**	**CNS**	**Copy Number (CN)**
TSC1	P1B	AMP	2.6
TSC2	P1B	AMP	2.6
USH2A	P1B	DEL	1.3
VHL	M1A	AMP	3.7
VHL	M1B	H.AMP	4.5
VHL	P1A	AMP	3.6
VHL	P1B	DEL	1.3
WT1	M1A	DEL	1.4
WT1	P1A	DEL	1.4
ZNF238	P1B	DEL	1.3
ZNF536	P1B	AMP	2.6

Supplementary Table 5: Sample identifiers and distances between multi-region samples.

Primary Site	History	Sample	Description	Tissue	Distance
Larynx	New Primary (Previously Untreated)	P1A	Primary Tumor	A	A to B 2.1 cm
		P1B	Primary Tumor	B	
		M1A	Met LN	A	A to B 2.4 cm
		M1B	Met LN	B	
		N1	Buffy coat		
Oral Tongue	Recurrent Tumor (Previously Treated with Cisplatin and Radiation	P2A	Primary Tumor	A	A to B 3.7 cm
		P2B	Primary Tumor	B	
		M2A	Met LN	A	
		N2	Buffy coat		
Floor-of-Mouth	New Primary (Previously Untreated)	P3A	Primary Tumor	A	A to B 1.5 cm
		P3B	Primary Tumor	B	
		M3A	Met LN	A	
		N3	Buffy coat		
Oral Tongue	New Primary (Previously Untreated)	P4A	Primary Tumor	A	A to B 5 mm
		P4B	Primary Tumor	B	
		N4	Buffy coat		
Oral Tongue	New Primary (Previously Untreated)	P5A	Primary Tumor	A	A to B 10mm
		P5B	Primary Tumor	B	B to C 15 mm
		P5C	Primary Tumor	C	
		M5A	Met LN	A	5 mm
		M5B	Met LN	B	
		N5	Buffy coat		
Oral Tongue	New Primary (Previously Untreated)	P6A	Primary Tumor	A	A to B 7mm
		P6B	Primary Tumor	B	B to C 8 mm
		P6C	Primary Tumor	C	
		M6A	Met LN	A	12 mm
		M6B	Met LN	B	
		N6	Buffy coat		
Larynx	New Primary (Previously Untreated)	P7A	Primary Tumor	A	A to B 1 cm
		P7B	Primary Tumor	B	
		N7	Buffy coat		

Supplementary Table 6: List of 202 clinically relevant cancer genes that were sequenced in this study.

Symbol	Chromosome region	Name
MTOR	chr1:11166589-11322608	mammalian target of rapamycin complex 1
NRAS	chr1:115247079-115259515	neuroblastoma RAS viral (v-ras) oncogene homolog
NOTCH2	chr1:120454178-120612276	Notch homolog 2
FLG	chr1:152274651-152297679	filaggrin
IL6R	chr1:154377669-154440188	interleukin 6 receptor
SPTA1	chr1:158580496-158656506	spectrin alpha, erythrocytic 1
SPEN	chr1:16174359-16266950	spen homolog, transcriptional regulator
DDR2	chr1:162602228-162750237	discoidin domain receptor tyrosine kinase 2
PAPPA2	chr1:176432307-176811968	pappalysin 2
HMCN1	chr1:185703683-186160085	hemicentin 1
USH2A	chr1:215796236-216596738	usher syndrome 2A
RYR2	chr1:237205702-237997288	regulatory factor X, 2 (influences HLA class II expression)
ZNF238	chr1:244214561-244220776	zinc finger protein 238
ARID1A	chr1:27022522-27108601	AT rich interactive domain 1A (SWI-like)
CSMD2	chr1:33979609-34630875	CUB and Sushi multiple domains 2
MPL	chr1:43803475-43820134	myeloproliferative leukemia virus oncogene
JAK1	chr1:65298906-65432187	Janus kinase 1
ERCC3	chr2:128014866-128051752	excision repair cross-complementing rodent repair deficiency, compl group 3
LRP1B	chr2:140988996-142889270	low density lipoprotein receptor-related protein 2
LRP2	chr2:169983620-170219122	low density lipoprotein receptor-related protein 3
ITGA4	chr2:182321619-182402466	integrin alpha 4 (antigen CD49D subunit of VLA-4 receptor)
CASP8	chr2:202122754-202152434	caspase 8, apoptosis-related cysteine peptidase
IDH1	chr2:209100954-209119806	isocitrate dehydrogenase 1 (NADP+), soluble
PIKFYVE	chr2:209130991-209223474	protein phosphatase 2A activator, regulatory subunit 4
IRS1	chr2:227596034-227663506	insulin receptor substrate 1
DNMT3A	chr2:25455846-25564774	DNA (cytosine-5-)-methyltransferase 3 alpha
ALK	chr2:29415641-30144432	anaplastic lymphoma kinase (Ki-1)
SOS1	chr2:39208692-39347604	son of sevenless homolog 1
EML4	chr2:42,396,490-42,559,686	echinoderm microtubule associated protein like 4
MSH2	chr2:47630263-47710360	mutS homolog 2 (E. coli)
MSH6	chr2:48,010,221-48,034,084	mutS homolog 6 (E. coli)
VHL	chr3:10183319-10193744	von Hippel-Lindau syndrome gene
RAF1	chr3:12625102-12705700	v-raf-1 murine leukemia viral oncogene homolog 1
FOXL2	chr3:138,663,067-138,665,982	forkhead box L2
ATR	chr3:142168078-142297668	ataxia telangiectasia and Rad3 related

Symbol	Chromosome region	Name
MECOM	chr3:168801287-169381563	MDS1 and EVI1 complex locus
PIK3CA	chr3:178866311-178952495	phosphoinositide-3-kinase, catalytic, alpha polypeptide
ETV5	chr3:185764108-185826901	ets variant 5
TGFBR2	chr3:30647994-30735631	transforming growth factor, beta receptor II
MLH1	chr3:37034979-37092335	E.coli MutL homolog gene
MYD88	chr3:38179969-38184510	myeloid differentiation primary response gene (88)
CTNNB1	chr3:41240942-41281939	catenin (cadherin-associated protein), beta 1
SETD2	chr3:47057900-47205467	SET domain containing 2
PBRM1	chr3:52,579,368-52,713,739	polybromo 1
BAP1	chr3:52435027-52444009	BRCA1 associated protein-1 (ubiquitin carboxy-terminal hydrolase)
MITF	chr3:69788633-70017486	microphthalmia-associated transcription factor
EPHA3	chr3:89156674-89531282	EPH receptor A3
TET2	chr4:106067943-106200958	tet oncogene family member 2
CRIPAK	chr4:1385340-1389782	cysteine-rich PAK1 inhibitor
FBXW7	chr4:153242411-153456185	F-box and WD-40 domain protein 7 (archipelago homolog, Drosophila)
FGFR3	chr4:1795039-1810599	fibroblast growth factor receptor 3
WHSC1	chr4:1873123-1983933	Wolf-Hirschhorn syndrome candidate 1
PDGFRA	chr4:55095264-55164411	platelet-derived growth factor, alpha-receptor
KIT	chr4:55524095-55606879	kallikrein-related peptidase 2
KDR	chr4:55944427-55991762	vascular endothelial growth factor receptor 2
LPHN3	chr4:62362839-62938167	latrophilin 3
APC	chr5:112073556-112181935	adenomatous polyposis of the colon gene
CSF1R	chr5:149432855-149492935	colony stimulating factor 1 receptor
PDGFRB	chr5:149493403-149535422	platelet-derived growth factor receptor, beta polypeptide
GABRA6	chr5:161112658-161129598	GABA A receptor, alpha 6
NPM1	chr5:170814798-170837887	nucleophosmin (nucleolar phosphoprotein B23, numatrin)
FGFR4	chr5:176513921-176525124	fibroblast growth factor receptor 4
NSD1	chr5:176560833-176727213	nuclear receptor binding SET domain protein 1
FLT4	chr5:180028507-180076624	fms-related tyrosine kinase 4
CDH10	chr5:24487210-24644911	cadherin 10, type 2
ADAMTS12	chr5:33,527,287-33,892,124	ADAM metallopeptidase with thrombospondin type 1 motif, 12
HEATR7B2	chr5:40998123-41071444	HEAT repeat family member 7B2
MAP3K1	chr5:56110900-56191976	mitogen-activated protein kinase kinase kinase 1
PIK3R1	chr5:67522118-67597647	phosphoinositide-3-kinase, regulatory subunit 1 (alpha)
PRDM1	chr6:106534195-106557814	PR domain containing 1, with ZNF domain
TNFAIP3	chr6:138188581-138204445	tumor necrosis factor, alpha-induced protein 3
ESR1	chr6:152163859-152424408	estrogen Receptor 1

Symbol	Chromosome region	Name
SYNE1	chr6:152442823-152958534	spectrin repeat containing, nuclear envelope 1
MAP3K4	chr6:161412822-161538416	mitogen-activated protein kinase kinase kinase 4
DDR1	chr6:30856465-30867931	discoidin domain receptor tyrosine kinase 1
NOTCH4	chr6:32162621-32191844	notch4
DAXX	chr6:33,286,336-33,290,793	death-domain associated protein
PKHD1	chr6:51480145-51952423	polycystic kidney and hepatic disease 1 (autosomal recessive)
BAI3	chr6:69345632-70099402	brain-specific angiogenesis inhibitor 3
MDN1	chr6:90353231-90529442	MDN1, midasin homolog
RELN	chr7:103112233-103629963	reelin
PIK3CG	chr7:106505924-106547585	phosphoinositide-3-kinase, catalytic, gamma polypeptide
PPP1R3A	chr7:113516882-113559082	protein phosphatase 1, regulatory (inhibitor) subunit 3A
MET	chr7:116312459-116438439	met proto-oncogene (hepatocyte growth factor receptor)
SMO	chr7:128828713-128853383	smoothened homolog (Drosophila)
BRAF	chr7:140433815-140624564	v-raf murine sarcoma viral oncogene homolog B1
PRSS1	chr7:142457319-142460927	protease, serine, 1 (trypsin 1)
EZH2	chr7:148504475-148581414	cer of zeste homolog 2 (Drosophila)
MLL3	chr7:151832012-152133090	myeloid/lymphoid or mixed-lineage leukemia 3
HDAC9	chr7:18535885-19036984	histone deacetylase 9
CARD11	chr7:2945769-3083579	caspase recruitment domain family, member 11
IKZF1	chr7:50344378-50472796	interleukin 21 receptor
EGFR	chr7:55086725-55275030	epidermal growth factor receptor
ELN	chr7:73442427-73484234	elastin
HGF	chr7:81331445-81399452	hepatocyte growth factor
PCLO	chr7:82383321-82792197	piccolo (presynaptic cytomatrix protein)
CDK6	chr7:92234237-92465941	cyclin-dependent kinase 6
RIMS2	chr8:104512976-105265451	regulating synaptic membrane exocytosis 2
PKHD1L1	chr8:110374706-110543499	polycystic kidney and hepatic disease 1 (autosomal recessive)-like 1
CSMD3	chr8:113235161-114449242	CUB and Sushi muliple domain 3
COL14A1	chr8:121137352-121384266	collagen, type XIV, alpha 1
FAM135B	chr8:139142268-139509065	family with sequence similarity 135, member B
PTK2	chr8:141668502-142011332	PTK2 protein tyrosine kinase 2
CSMD1	chr8:2792876-4852328	CUB and Sushi muliple domain 1
FGFR1	chr8:38268657-38326352	FGFR1 oncogene partner (FOP)
KCNB2	chr8:73449626-73850582	potassium voltage-gated channel, Shab-related subfamily, member 2
RUNX1T1	chr8:92971152-93088365	runt-related transcription factor 1
PPP2R4	chr9:131873244-131911223	protein phosphatase 2A activator, regulatory subunit 4
ABL1	chr9:133,710,831-133,763,060	c-abl oncogene 1, non-receptor tyrosine kinase

Symbol	Chromosome region	Name
TSC1	chr9:135766735-135820020	tuberous sclerosis 1
NOTCH1	chr9:139388897-139440238	Notch homolog 1, translocation-associated (Drosophila) (TAN1)
CDKN2A	chr9:21967752-21994490	cyclin-dependent kinase inhibitor 2A (p16(INK4a)) gene
PAX5	chr9:36838531-37034476	paired box gene 5 (B-cell lineage specific activator protein)
JAK2	chr9:4985245-5128182	Janus kinase 3
GNAQ	chr9:80335200-80646192	guanine nucleotide binding protein (G protein), q polypeptide
SYK	chr9:93564012-93660833	spleen tyrosine kinase
PTCH1	chr9:98205266-98270831	Homolog of Drosophila Patched gene1
NFKB2	chr10:104154339-104162280	nuclear factor of kappa light polypeptide gene enhancer in B-cells 2
FGFR2	chr10:123237845-123357972	fibroblast growth factor receptor 2
RET	chr10:43572517-43625795	ret proto-oncogene
PCDH15	chr10:55580860-56561051	protocadherin 1
GATA3	chr10:8096667-8117162	guanine monphosphate synthetase
PTEN	chr10:89623195-89728531	phosphatase and tensin homolog gene
CYP2C19	chr10:96522463-96612670	cytochrome P450, family 2, subfamily C, polypeptide 19
ATM	chr11:108093559-108239826	ataxia telangiectasia mutated
CBL	chr11:119076990-119178858	Cas-Br-M (murine) ecotropic retroviral transforming sequence
CHEK1	chr11:125496312-125525639	CHK1 checkpoint homolog (S. pombe)
WT1	chr11:32409325-32457087	Wilms tumour 1 gene
HRAS	chr11:532243-535550	v-Ha-ras Harvey rat sarcoma viral oncogene homolog
MEN1	chr11:64570996-64578188	multiple endocrine neoplasia type 1 gene
FAT3	chr11:92,085,262-92,629,633	fat tumor suppressor 3
PTPN11	chr12:112856536-112947716	protein tyrosine phosphatase, non-receptor type 11
HNF1A	chr12:121416549-121440312	transcription factor 1, hepatic (HNF1)
KRAS	chr12:25358180-25403854	v-Ki-ras2 Kirsten rat sarcoma 2 viral oncogene homolog
AKAP3	chr12:4,724,677-4,754,358	A kinase (PRKA) anchor protein 3
MLL2	chr12:49412762-49449107	myeloid/lymphoid or mixed-lineage leukemia 2
ACVR1B	chr12:52345486-52390859	activin A receptor, type IB
ERBB3	chr12:56,473,892-56,497,127	v-erb-b2 erythoblastic lekemia viral oncogene homolog 3 (avian)
LRP1	chr12:57,522,282-57,607,123	low density lipoprotein receptor-related protein 1B
CDK4	chr12:58142005-58146164	cyclin-dependent kinase 4
NAV3	chr12:78225069-78606788	neuron navigator 3
ERCC5	chr13:103459496-103524748	excision repair cross-complementing rodent repair deficiency, comp group 6
FLT3	chr13:28577412-28674729	fms-related tyrosine kinase 3
FLT1	chr13:28874483-29069265	fms-related tyrosine kinase 1 (VEGF/vascular permeability factor receptor)
BRCA2	chr13:32889617-32973809	familial breast/ovarian cancer gene 2
RB1	chr13:48877883-49056024	retinoblastoma gene

Symbol	Chromosome region	Name
TBC1D4	chr13:75858809-76056250	TBC1 domain family, member 4
AKT1	chr14:105235689-105262080	v-akt murine thymoma viral oncogene homolog 1
SYNE2	chr14:64319683-64693165	spectrin repeat containing, nuclear envelope 2
TSHR	chr14:81421869-81612646	thyroid stimulating hormone receptor
GABRB3	chr15:26788695-27018251	GABA A1 receptor, beta 3
RAD51	chr15:40987327-41024354	RAD51 homolog
MAP2K1	chr15:66679211-66783881	mitogen-activated protein kinase kinase 1
IDH2	chr15:90627214-90645708	isocitrate dehydrogenase 2 (NADP+), soluble
IGF1R	chr15:99192761-99507758	insulin-like growth factor 1 receptor
ERCC4	chr16:14014014-14046205	excision repair cross-complementing rodent repair deficiency, compl group 4
TSC2	chr16:2097990-2138712	tuberous sclerosis 2
PALB2	chr16:23614483-23652678	partner and localizer of BRCA2
CD19	chr16:28943260-28950661	CD19 molecule
CREBBP	chr16:3775058-3930121	CREB binding protein (CBP)
CYLD	chr16:50775961-50835846	familial cylindromatosis gene
CDH11	chr16:64980685-65155919	cyclin-dependent kinase 11
CDH1	chr16:68771195-68869444	cadherin 1, type 1, E-cadherin (epithelial) (ECAD)
HYDIN	chr16:70841290-71264569	hydrocephalus inducing homolog
MAP2K4	chr17:11924135-12047050	mitogen-activated protein kinase kinase 4
NCOR1	chr17:15935259-16118845	nuclear receptor corepressor 1
NF1	chr17:29,421,995-29,704,694	neurofibromatosis type 1 gene
HNF1B	chr17:36046435-36105096	HNF1 homeobox B
ERBB2	chr17:37856254-37884914	v-erb-b2 erythroblastic leukemia viral oncogene homolog 2
TOP2A	chr17:38544798-38574169	topoisomerase II
BRCA1	chr17:41196313-41277500	familial breast/ovarian cancer gene 1
SPOP	chr17:47676248-47755525	speckle-type POZ protein
TP53	chr17:7571720-7590863	tumor protein p53
RNF213	chr17:78313726-78370078	ring finger protein 213
AURKB	chr17:8108050-8113883	aurora kinase B
SMAD4	chr18:48556583-48611409	SMAD family member 4
LAMA1	chr18:6941888-7117813	laminin, alpha 1
SMARCA4	chr19:11071598-11172959	SWI/SNF related, matrix assoc, actin dep reg of chrom, subfamily a, member 4
STK11	chr19:1205798-1228434	serine/threonine kinase 11 gene (LKB1)
NOTCH3	chr19:15270445-15311792	notch 3
CPAMD8	chr19:17003763-17137625	C3 and PZP-like, alpha-2-macroglobulin domain containing 8
JAK3	chr19:17935595-17958841	Janus kinase 3
ZNF536	chr19:30863328-31048965	zinc finger protein 536

Symbol	Chromosome region	Name
GNA11	chr19:3094408-3121452	guanine nucleotide binding protein (G protein), alpha 11 (Gq class)
CEBPA	chr19:33790842-33793430	CCAAT/enhancer binding protein (C/EBP), alpha
TGFb1	chr19:41836651-41859816	transforming growth factor, beta 1
PPP2R1A	chr19:52693191-52729670	protein phosphatase 2, regulatory subunit A, alpha
ASXL1	chr20:30946153-31027121	additional sex combs like 1
TOP1	chr20:39657462-39753124	topoisomerase (DNA) I
AURKA	chr20:54944445-54967351	aurora kinas A
GNAS	chr20:57414795-57486249	guanine nucleotide binding protein (G protein), alpha stimulating activ polyp 1
RUNX1	chr21:36160099-36421595	runt-related transcription factor 1 (AML1)
SMARCB1	chr22:24129150-24176704	SWI/SNF related, matrix assoc, actin dep reg of chrom, subfamily b, member 1
CHEK2	chr22:29083731-29137822	CHK2 checkpoint homolog
NF2	chr22:29999545-30094583	neurofibromatosis type 2 gene
EP300	chr22:41488614-41576080	300 kd E1A-Binding protein gene
KDM6A	chrX:44732423-44971843	vascular endothelial growth factor receptor 2
ARAF	chrX:47420578-47431319	v-raf murine sarcoma 3611 viral oncogene homolog
GATA1	chrX:48,644,982-48,652,715	GATA binding protein 3
FAM123B	chrX:63404998-63425624	family with sequence similarity 123B (FAM123B)
AR	chrX:66763874-66944119	androgen receptor
ATRX	chrX:76760359-77041719	alpha thalassemia/mental retardation syndrome X-linked
